# Magnesium Depletion Score and Mortality in Individuals with Metabolic Dysfunction Associated Steatotic Liver Disease over a Median Follow-Up of 26 Years

**DOI:** 10.3390/nu17020244

**Published:** 2025-01-10

**Authors:** Lei Fan, Xiangzhu Zhu, Xinyuan Zhang, Shakirat Salvador, Xuehong Zhang, Martha J. Shrubsole, Manhal J. Izzy, Qi Dai

**Affiliations:** 1 Vanderbilt Memory and Alzheimer’s Center, Vanderbilt University School of Medicine, Nashville, TN 37203, USA; lei.fan.1@vumc.org; 2 Department of Neurology, Vanderbilt University Medical Center, Nashville, TN 37203, USA; 3 Division of Epidemiology, Vanderbilt Epidemiology Center, Department of Medicine, Vanderbilt University Medical Center, Vanderbilt University School of Medicine, Nashville, TN 37203, USA; martha.shrubsole@vanderbilt.edu; 4 Channing Division of Network Medicine, Department of Medicine, Brigham and Women’s Hospital and Harvard Medical School, Boston, MA 02115, USA; hpxzh@channing.harvard.edu; 5 Division of Gastroenterology, Hepatology and Nutrition, Department of Medicine, Vanderbilt University Medical Center, Nashville, TN 37232, USA; shakirat.salvador@vumc.org; 6 Department of Nutrition, Harvard T.H. Chan School of Public Health, Boston, MA 02115, USA; xuehong.zhang@yale.edu; 7 Yale School of Nursing, Orange, CT 06477, USA; 8 International Epidemiology Field Station, Vanderbilt University Medical Center, Nashville, TN 37232, USA

**Keywords:** metabolic dysfunction associated steatotic liver disease, magnesium depletion score, all-cause mortality, cardiovascular disease mortality, cancer mortality, magnesium intake

## Abstract

Metabolic dysfunction associated steatotic liver disease (MASLD) has been associated with increased risks of all-cause and cardiovascular disease (CVD) mortality. Identification of modifiable risk factors that may contribute to higher risks of mortality could facilitate targeted and intensive intervention strategies in this population. This study aims to examine whether the magnesium depletion score (MDS) is associated with all-cause and CVD mortality among individuals with MASLD or metabolic and alcohol associated liver disease (MetALD). Methods: A total of 3802 participants with MASLD or MetALD were followed up over a median of 26 years in the National Health and Nutrition Examination Survey (NHANES) III cohort. The MDS was calculated by aggregating four factors influencing the reabsorption capability of the kidneys. The associations between MDS and all-cause, CVD, and cancer mortality were quantified using Cox proportional hazard regression models. Results: In the combined MASLD + MetALD cohort, a higher MDS (>2) was associated with increased all-cause mortality (HR, 2.52; 95%CI, 1.77–3.61; *p*-trend < 0.0001) and CVD mortality (HR, 3.01; 1.87–4.86; *p*-trend < 0.0001) compared to MDS = 0; this association became stronger among participants who did not meet the estimated average requirement level of Mg intake (2.72; 1.69–4.37; *p*-trend = 0.0014) and those with a Fibrosis-4 index (FIB-4) < 1.3 (2.95; 1.69–5.15; *p*-trend = 0.0006). Conclusions: In individuals with MASLD or MetALD, higher MDS, indicative of worse global Mg status, was associated with an increased risk of all-cause and CVD mortality. Correcting global Mg deficiency in high-risk MASLD/MetALD patients may have long-term health benefits.

## 1. Introduction

Nonalcoholic fatty liver disease (NAFLD)—which encompasses a series of progressive liver diseases from steatosis, nonalcoholic steatohepatitis (NASH), fibrosis, and cirrhosis—affects a quarter to a third of the adult population worldwide [1,2]. While liver-related mortality only accounts for 7% of total deaths among those with NAFLD [3,4], cardiovascular disease (CVD) followed by extrahepatic malignancy remains the leading cause of death in individuals with NAFLD [1]. The metabolic dysfunction associated steatotic liver disease (MASLD) and metabolic and alcohol associated liver disease (MetALD) were newly proposed to minimize stigma and broaden conditions with various etiologies of steatosis in the absence of significant alcohol use [5]. Individuals with MASLD or MetALD are at an increased risk of developing incident CVD [6]. Furthermore, two nationwide studies found that participants with MASLD or MetALD had 1.2- to 1.5-fold higher all-cause mortality than those without steatotic liver disease (SLD) [7,8]. Identifying patients with MASLD/MetALD at higher risk of mortality could facilitate risk stratification and targeted intervention strategies in this population.

Low magnesium (Mg) status has been linked to both chronic systemic inflammation and insulin resistance, which leads to increased vulnerability to liver damage and deterioration of liver disease [9]. We previously reported that a higher intake of Mg was associated with reduced odds of both fatty liver disease [10] and risk of mortality due to liver diseases, particularly among alcohol drinkers and those with hepatic steatosis [11]. Subsequent studies also associated either Mg intake or serum Mg with the risk of NAFLD, liver fibrosis, and hepatocellular carcinoma among patients with NAFLD [12,13,14,15]. However, no previous study has examined Mg status in relation to all-cause, CVD, and cancer-related mortality among individuals with MASLD or MetALD.

We previously developed and validated an instrument tool, the Mg depletion score (MDS), in predicting body Mg status [16]. The MDS incorporated pathophysiological factors influencing the kidneys’ reabsorption capability and performed better than both serum Mg and Mg intake [16,17]. Furthermore, we found that a higher MDS was associated with an increased risk of all-cause and CVD mortality as well as systemic inflammation, particularly among those who did not meet the estimated average requirement (EAR) of Mg intake, assessed by 24-h dietary recall [16]. This study aimed to examine whether the MDS in combination with Mg intake can be used to risk stratify all-cause, CVD, and cancer-related mortality among individuals with MASLD or MetALD in the National Health and Nutrition Examination Survey (NHANES) III cohort, a nationally representative sample of the United States (U.S.) general population.

## 2. Materials and Methods

### 2.1. Study Population

The NHANES III, conducted in the U.S. from 1988 to 1994, was designed to investigate the health and nutritional status of the U.S. general population. It consists of interviews, medical examinations, and laboratory data collected from a complex multistage, stratified, clustered probability sample representative of the national non-institutionalized population. This study was approved by the institutional review board of the Centers for Disease Control and Prevention (CDC), and all participants provided written informed consent. Publicly available data from de-identified participants were used in this study. In total, 33,994 individuals aged 2 months and older participated in the survey, among whom 14,797 adults aged 20–74 years underwent ultrasound examinations. Ultrasound examination was conducted using a Toshiba Sonolayer SSA-90A and Toshiba video recorder (Tokyo, Japan) [18]. To confirm the presence of fat within the hepatic parenchyma, archived gallbladder ultrasound video images were reviewed and assessed by three ultrasound readers trained by a board-certified radiologist specializing in hepatic imaging. After excluding 941 participants with no available images or grading and 8840 participants with normal findings, 5016 participants were confirmed to have hepatic steatosis. Among the 5016 participants with hepatic steatosis, individuals with missing alcohol consumption data or excessive alcohol consumption that met the criteria of alcohol-related liver disease (ALD) (>50 g per day for women and >60 g per day for men, *n* = 710) [5], viral hepatitis (*n* = 496), or missing MDS information (*n* = 8) were further excluded, leaving 3802 participants for analysis, among whom 3560 and 242 participants were identified as MASLD and MetALD, respectively.

### 2.2. Metabolic-Dysfunction-Associated Steatotic Liver Disease (MASLD)

Pure MASLD was defined as the presence of ultrasound-confirmed hepatic steatosis, with alcohol consumption below 20 g per day in women and 30 g per day in men [5], and the presence of at least one of the following cardiometabolic criteria [19]: (1) overweight or obesity with body mass index (BMI) ≥ 25 kg/m^2^, Asian: BMI ≥ 23 kg/m^2^ or waist circumference > 94 cm in men and 80 cm in women; (2) diabetes—glycated hemoglobin (HbA1c) ≥ 5.7%, or fasting plasma glucose ≥ 5.6 mmol/L, or 2-h post-load glucose level > 7.8 mmol/L, or previous type 2 diabetes diagnosis, or currently taking antidiabetics; (3) hypertension (blood pressure ≥ 130/85 mmHg or under anti-hypertension treatment); (4) plasma high-density lipoprotein cholesterol (HDL-C) < 1.0 mmol/L for men and <1.3 mmol/L for women; or (5) plasma triglycerides (TG) ≥ 1.70 mmol/L or under lipid-lowering treatment.

### 2.3. Metabolic- and Alcohol-Related/Associated Liver Disease (MetALD)

The MetALD category was defined as the presence of ultrasound-confirmed hepatic steatosis and at least one of the above-mentioned cardiometabolic criteria, with alcohol consumption of 20–50 g per day in women, 30–60 g per day in men [5].

### 2.4. MDS and Dietary Assessment of Mg

The MDS was previously validated [16] and replicated in several studies [20,21,22,23,24,25,26,27,28,29]. By aggregating four factors, the total MDS ranged from 0 to 5, with higher scores indicating more severe Mg depletion: (1) diuretic use (current use for 1 point); (2) proton pump inhibitor (PPI) use (current use for 1 point); (3) kidney function [60 mL/(min·1.73 m^2^) ≤ estimated glomerular filtration rate (eGFR) < 90 mL/(min·1.73 m^2^) for 1 point, eGFR < 60 mL/(min·1.73 m^2^) for 2 points]; and (4) alcohol drinking (current heavy drinker for 1 point). Heavy drinkers were defined as >1 drink per day for women and >2 drinks per day for men [16,30,31]. A standard drink was defined as a drink with 14 g (0.6 fluid ounces) of pure alcohol [32]. Daily dietary intakes were calculated from one 24-h dietary recall and 30-day supplement interviews, which were obtained at baseline and were described in detail elsewhere [33]. We only included dietary recall data with a status of ‘reliable’ in the current analysis. Supplemental intake of Mg was collected from responses to a dietary supplement survey questionnaire. The total Mg intake was calculated by summing the intake from the diet and supplements. The age- and sex-specific estimated average requirement (EAR) was used to classify Mg intake: 330 mg per day for men aged 19–30 years; 350 mg per day for men aged 31 years and older; 255 mg per day for women aged 19–30 years; and 265 mg per day for women aged 31 years and older [34]. These values were set to meet the requirements of half of the healthy individuals in these age- and sex-specific groups.

### 2.5. Covariates

The covariates were selected by an a priori review of sociodemographic and lifestyle factors related to Mg status and mortality outcomes. We adjusted for covariates including age (continuous), sex (men, women), race and ethnicity (Non-Hispanic White, Non-Hispanic Black, Hispanic, or other), education level (lower than high school education, high school diploma or general education development, and some college or above), income-to-poverty ratio (PIR; PIR ≤ 1, PIR 1–3, and PIR > 3), cigarette smoking (never, former, and current), alcohol drinking status (never, former, and current), physical activity (inactive, insufficiently active, recommended activity), BMI (continuous), and daily intakes of total energy (continuous). Sociodemographic factors, such as PIR, were related to differences in nutritional status, disease risk, and mortality. Lifestyle factors such as alcohol consumption, smoking, and physical activity were associated with nutrient absorption and utilization, as well as disease risk and mortality. Although the current heavy alcohol intake was considered in the MDS algorithm, other alcohol drinking statuses, such as current-drinkers and former-drinkers, may act as residual confounding factors. As a result, we also adjusted for alcohol drinking status as a covariate (never, former, and current).

### 2.6. Outcomes: All-Cause Mortality, CV Mortality, and Cancer-Related Mortality

Vital status and cause of death were identified using probabilistic linkage to the National Death Index (NDI) through 31 December 2019. The cause of death coding for all deaths occurring prior to 1999 adhered to the 9th revision of the International Statistical Classification of Diseases, Injuries, and Causes of Death (ICD-9) guidelines, while all deaths after 1998 followed the 10th revision of the International Statistical Classification of Diseases, Injuries, and Causes of Death (ICD-10) guidelines. The major outcomes included all-cause death, death from CVD (ICD-10 codes I00–I09, I11, I13, I20–I51, I60–I69, I70, and I71–I78), and death from cancer (ICD-10 codes C00–C97).

### 2.7. Statistical Analyses

The baseline characteristics of study participants were compared across MDS (0, 1, 2, >2) using a survey regression model for continuous variables and Rao–Scott chi-square tests for categorical variables. Hazard ratios (HRs) and 95% confidence intervals (CIs) were estimated using Cox proportional hazard regression models for associations between MDS and the risks of all-cause, CVD, and cancer mortality. Three models were used to estimate the HRs (and 95%CIs) associated with MDS categories. Model 1: adjusted for age, sex, and race/ethnicity. Model 2: model 1 + additionally adjusted for education level, PIR, cigarette smoking status, alcohol drinking status, physical activity, BMI, total energy intake, and Mg intake. Model 3: model 2 + additionally adjusted for Fibrosis-4 index (FIB4) [35]. Two stratified analyses were further conducted to examine whether the associations between MDS and risks of all-cause, CVD, and cancer mortality differed by the EAR level of Mg intake or FIB4, respectively. We conducted the following sensitivity analyses in both the combined MASLD + MetALD cohort and pure MASLD cohorts: (1) to examine the MDS in association with the risk of all-cause mortality by excluding participants who died within the first year of follow-up; (2) to examine the MDS in relation to CVD mortality by excluding participants with a history of CVD at baseline; (3) to examine the MDS in association with cancer mortality by excluding participants with a history of cancer at baseline; and (4) to examine the MDS in relation to all-cause, CVD, and cancer mortality by additionally adjusting for comorbidities of obesity, diabetes, hypertension, and dyslipidemia based on Model 3. All analyses accounted for NHANES sampling weights, non-responses, clusters, and strata. All analyses were conducted using SAS version 9.4 (SAS Institute, Cary, NC, USA), and all hypotheses testing were 2-sided, with *p* < 0.05 indicating statistical significance.

## 3. Results

The baseline characteristics of the participants with MASLD or MetALD according to MDS are presented in Table 1. In the combined MASLD + MetALD cohort, compared with participants with MDS = 0, participants with MDS > 2 were more likely to be female (weighted percentage, 66.6% vs. 47.3%), older (weighted mean [SD] age, 64.2 [1.1] vs. 39.7 [0.4]), Non-Hispanic White (92.4% vs. 65.9%), have lower education levels (less than high school, 31.3% vs. 29.3%), have a higher PIR (PIR > 1, 90.3% vs. 82.4%), be physically inactive (29.5% vs. 19.9%), have a lower total energy intake (weighted mean [SD], 1536 [130] vs. 2243 [39]), higher BMI (weighted mean [SD], 32.1 [0.8] vs. 30.0 [0.4]), higher FIB4 (1.5 [0.1] vs. 0.8 [0.0]), and were less likely to be a current smoker (11.7% vs. 27.8%). The baseline characteristics of the participants with pure MASLD according to MDS are presented in Supplemental Table S1.

### 3.1. All-Cause, CVD, and Cancer Mortality for MASLD or MetALD

The current study included 3802 participants with MASLD or MetALD (Table 2) and 3560 participants with pure MASLD (Table 3). In the combined MASLD + MetALD cohort, 1638 all-cause deaths occurred among 3802 participants over a median follow-up interval of 26 years, of which 542 were deaths from CVD and 360 from cancer. In the pure MASLD cohort, 1544 all-cause deaths occurred among 3560 pure MASLD participants over a median follow-up interval of 26 years, among whom 517 died from CVD and 338 from cancer.

### 3.2. MDS in Relation to All-Cause, CVD, and Cancer Mortality in the Combined Cohort of MASLD + MetALD

In the combined cohort of MASLD + MetALD, a higher MDS was significantly associated with an over two-fold increased risk of all-cause mortality in the fully adjusted model (HR, 2.52; 95%CI, 1.77–3.61; *p* for trend < 0.0001) compared with participants with MDS > 2 to MDS = 0 (Table 2). When stratified by the EAR level of Mg intake, the associations remained significant in both strata. However, it became slightly stronger among participants who did not meet the EAR level of Mg intake (HR, 2.72; 95%CI, 1.69–4.37; *p* for trend = 0.001) and marginally attenuated among those who consumed Mg above the EAR level (HR, 2.29; 95%CI, 1.36–3.85; *p* for trend = 0.0003) (*p*-interaction = 0.93).

In the combined cohort of MASLD + MetALD, a higher MDS was significantly associated with over three-fold increased risk of CVD mortality in the fully adjusted model (HR, 3.01; 95%CI, 1.87–4.86; *p* for trend < 0.0001) comparing participants with MDS > 2 to MDS = 0 (Table 2). In the stratified analysis by the EAR level of Mg intake, the associations remained significant and became stronger only among participants who did not meet the EAR level of Mg intake (HR, 3.77; 95%CI, 1.88–7.55; *p* for trend = 0.0004), whereas we did not observe a significant association among those who consumed Mg above the EAR level (*p*-interaction = 0.93). We did not observe any associations between MDS and cancer mortality in the combined cohort of MASLD + MetALD (Supplemental Table S8).

### 3.3. MDS in Relation to All-Cause, CVD, and Cancer Mortality in the Cohort of Pure MASLD

Similar associations were observed in the pure MASLD cohort, where a higher MDS was significantly associated with an over two-fold increased risk of all-cause mortality in the fully adjusted model (HR, 2.72; 95%CI, 1.75–4.22; *p* for trend < 0.0001) comparing participants with MDS > 2 to MDS = 0 (Table 3). When stratified by the EAR level of Mg intake, the associations remained significant in both strata; however, it became stronger among participants who did not meet the EAR level of Mg intake (HR, 2.97; 95%CI, 1.74–5.04; *p* for trend = 0.003), whereas it was attenuated among those who consumed Mg above the EAR level (HR, 2.24; 95%CI, 1.21–4.17; *p* for trend = 0.0003) (*p*-interaction = 0.71).

In the pure MASLD cohort, similar association patterns were observed for mortality due to CVD and in stratified analyses by EAR level of Mg intake. A higher MDS was significantly associated with about four-fold increased risk of CVD mortality in the fully adjusted model (HR, 3.86; 95%CI, 2.29–6.50; *p* for trend < 0.0001) comparing participants with MDS > 2 to MDS = 0 (Table 3). When stratified by the EAR level of Mg intake, the associations remained significant and became stronger among participants who did not meet the EAR level of Mg intake (HR, 4.65; 95%CI, 2.10–10.31; *p* for trend < 0.0001), while the association was attenuated among those who consumed Mg above the EAR level (HR, 3.02; 95%CI, 1.05–8.71; *p* for trend = 0.01) (*p*-interaction = 0.96). We did not observe any associations between MDS and cancer mortality in the pure MASLD cohort (Supplemental Table S9).

### 3.4. Stratified Analyses by FIB4 in the Combined Cohort of MASLD + MetALD

When stratified by FIB4 (FIB4 < 1.3 vs. FIB4 ≥ 1.3) in the combined cohort of MASLD + MetALD, the association between MDS and all-cause mortality became stronger among those with FIB4 < 1.3 in the fully adjusted model (HR, 2.95; 95%CI, 1.69–5.15; *p* for trend = 0.0006), whereas it became weaker in participants with FIB4 ≥ 1.3 (HR, 1.89; 95%CI, 1.15–3.10; *p* for trend = 0.04) (*p*-interaction = 0.57) (Table 4). For CVD mortality, a significant association was only present and strengthened in those with FIB4 < 1.3 (HR, 3.38; 95%CI, 1.77–6.44; *p* for trend < 0.0001) but not in participants with FIB4 ≥ 1.3 (*p*-interaction = 0.32).

### 3.5. Stratified Analyses by FIB4 in the Cohort of Pure MASLD

In stratified analysis by FIB4 (FIB4 < 1.3 vs. FIB4 ≥ 1.3) conducted in the cohort of pure MASLD, the association between MDS and all-cause mortality became stronger among those with FIB4 < 1.3 in the fully adjusted model (HR, 3.03; 95%CI, 1.53–6.00; *p* for trend = 0.001), while the association was not present in participants with FIB4 ≥ 1.3 (*p*-interaction = 0.29) (Table 5). Similar patterns were observed for CVD mortality. The significant association was solely observed and became stronger in those with FIB4 < 1.3 (HR, 4.25; 95%CI, 2.26–7.99; *p* for trend < 0.0001) but disappeared in participants with FIB4 ≥ 1.3 (*p*-interaction = 0.38).

### 3.6. Sensitivity Analyses

Consistent with the main results, sensitivity analyses conducted in the combined cohort of MASLD + MetALD and the cohort of pure MASLD showed similar associations for MDS with all-cause mortality overall and in subgroup analyses after excluding participants who died within the first year of follow-up (Supplemental Tables S2–S5). Sensitivity analyses for CVD mortality also led to similar association patterns as the main results after excluding participants with a history of CVD at baseline. Null associations remained for cancer mortality after excluding participants with a history of cancer at the baseline. We also conducted a sensitivity analysis by additionally adjusting for the comorbidities of obesity, diabetes, hypertension, and dyslipidemia based on model 3, and the results remained consistent with the main results (Supplemental Tables S6 and S7).

## 4. Discussion

This study, conducted in those with MASLD or MetALD from a nationally representative sample of U.S. adults over a median follow-up interval of 26 years, showed that a higher MDS, greater than two, was longitudinally associated with two- and three-fold increased risks for all-cause and CVD mortality, respectively. The associations were more prominent among participants who did not meet the EAR levels of Mg intake and those with a lower risk of advanced hepatic fibrosis (FIB4 < 1.3). To the best of our knowledge, this is the first study to establish a relationship between global Mg status and all-cause and CVD mortality among individuals with MASLD or MetALD.

MASLD is the most prevalent liver disease in the Western world and has been reported in over 76% of type 2 diabetes and 90% of obese individuals undergoing bariatric surgery [36]. Despite the recent Food and Drug Administration (FDA) conditional approval of resmetirom, lifestyle modifications such as nutritional changes and physical exercise remain widely recommended [37]. Long-term studies are needed to understand how modifiable risk factors impact MASLD or MetALD progression over time and, therefore, to facilitate the development of tools for risk stratification and precision-based intervention strategies in this population. It has been well-documented that CVD and extrahepatic malignancy are the leading causes of death in individuals with NAFLD [1]. Because the terms MASLD and MetALD have been recently introduced, relatively few studies have investigated modifiable factors that may contribute to MASLD-associated increases in all-cause and CVD mortality. While individuals with MASLD or MetALD had higher all-cause mortality than those without SLD [7,8], a recent study found that MASLD did not increase all-cause mortality compared to the non-SLD group in participants with cardiometabolic risk factors (CMRF), including abdominal obesity, dyslipidemia, impaired glycemic control, and hypertension. This suggests that CMRF, not SLD, is the main driver of all-cause and CVD mortality [38]. Mg deficiency has been consistently linked to CMRF and increased levels of inflammatory markers, which are risk factors for CVD. In addition to the risk of NAFLD [10], liver disease mortality [11], and HCC among those with NAFLD [15], previous studies have demonstrated that low Mg status measured by serum Mg or Mg intake was associated with an increased risk of prediabetes [10], type 2 diabetes, insulin resistance [39], and metabolic syndrome [40], which are diseases or conditions strongly linked to MASLD. Further, insulin resistance plays a key role in the initiation and progression of MASLD. Notably, there is a vicious cycle between Mg deficiency and insulin resistance, where insulin resistance induced by chronic Mg deficiency compromises cellular glucose utilization, exacerbating the loss of Mg in the kidneys and leading to further insulin resistance [41]. Moreover, Mg deficiency has been identified as a significant contributor to chronic low-grade inflammation, which is a risk factor for a variety of pathological conditions, including CVD and cancer [42,43]. The findings from previous studies evaluating Mg status and mortality have not been consistent. A systematic review and meta-analysis of prospective cohort studies found a significant inverse association between dietary Mg intake and mortality from all causes and cancer, while no significant association was observed between supplemental and total Mg intakes and deaths from all causes, CVD, and cancer [44]. Another prospective analysis conducted among 14,353 participants from the NHANES I Epidemiologic Follow-up Study (NHEFS) found that only extremely low concentrations of serum Mg (<0.70 mmol/L) were significantly associated with all-cause mortality, while marginal non-significant trends were observed for cancer and CVD mortality [45].

It is worth noting that these previous studies using either serum Mg or dietary Mg intake did not consider the kidneys’ reabsorption of Mg, which is essential in maintaining the homeostasis of body Mg [16]. In a 2021 report, we originally developed and validated that the MDS that incorporated pathophysiological factors influencing the kidneys’ reabsorption capability had a better performance of area under the receiver operating characteristic (ROC) curve (AUC) in predicting body Mg status than serum Mg and Mg intake [16]. We reported that a higher MDS was associated with an increased risk of all-cause and CVD mortality among individuals who did not meet the EAR level of Mg intake in the U.S. general population [16]. Following our report, several recent studies consistently revealed that a higher MDS was associated with risks of a wide spectrum of Mg deficiency-associated extrahepatic conditions [22,23,24,25,26,27,28,29], particularly in combination with Mg intake. The current study demonstrated that in both the combined MASLD + MetALD and pure MASLD cohorts, a higher MDS (>2) was associated with substantially increased risks of all-cause and CVD mortality, particularly when Mg intake was below the EAR. This is consistent with our original findings conducted in the U.S. general population [16]. We also observed that the association was primarily present in individuals with a lower risk of advanced hepatic fibrosis, suggesting that the MDS is more effective at risk stratifying all-cause and CVD mortality in the early stages of MASLD. We observed null associations with overall cancer mortality and in stratified analyses by EAR level of Mg intake or FIB4; however, further studies are warranted to eliminate the possibility of false-negative findings owing to insufficient statistical power.

### Strengths and Limitations

This study has several strengths. First, since the NHANES III cohort—from which individuals with MASLD or MetALD were derived—was a nationally representative sample of the U.S. population with a median follow-up interval of 26 years, selection bias was largely reduced. Second, when the survey data were collected, recall and interviewer biases were minimized because neither the participants nor the interviewers were aware of the hypothesis proposed in the current analysis. Finally, we conducted comprehensive sensitivity analyses, which showed results that were consistent with the main analysis. Additionally, the results are biologically plausible and are internally and externally consistent.

There were a few limitations to the current study. First, the four domains of information for calculating MDS and Mg intake were collected once at baseline, which may have fluctuated during the follow-up interval. In future studies, repeated measurements of the MDS and Mg intake may improve the estimates of body Mg status and enhance the analysis of associations. Although noninvasive and commonly used, ultrasound examination for identifying individuals with MASLD had only moderate diagnostic accuracy [46], and the sensitivity and specificity further decreased among obese patients with increasing BMI [47,48]. Therefore, the potential misclassification of MASLD diagnoses may increase the likelihood of a Type II error. Nevertheless, we observed substantially increased risks of all-cause and CVD mortality in the MASLD + MetALD and pure MASLD cohorts, indicating that MDS combined with Mg intake is a promising risk stratification tool for patients with MASLD or MetALD.

## 5. Conclusions

In individuals with MASLD or MetALD from a nationally representative sample of U.S. adults, a higher MDS—indicative of worse body Mg status—was associated with substantially increased risks of all-cause and CVD mortality. These associations were more pronounced in individuals who did not meet the EAR level of Mg intake or those with a lower risk of advanced hepatic fibrosis. The MDS is a promising tool that needs to be investigated for risk stratification and disease management of patients with MASLD or MetALD, preferably among individuals at the early stages of MASLD. Identifying MASLD/MetALD patients at high risk of Mg deficiency using the MDS, and correcting Mg deficiency through supplementation and dietary changes, may be important prevention strategies for reducing CVD mortality in MASLD/MetALD patients.

## Figures and Tables

**Table 1:** Baseline demographic and selected risk factors by MDS in participants with MASLD or MetALD in the NHANES III (MASLD + MetALD, *n* = 3802).

Characteristics	MDS = 0	MDS = 1	MDS = 2	MDS > 2	*p* Value
	*n* = 1931	*n* = 1408	*n* = 379	*n* = 84	
Age (year), Mean (SE)	39.7 (0.4)	49.7 (0.7)	58.4 (0.7)	64.2 (1.1)	<0.0001
Gender, *n* (%)					
Male	845 (52.7)	711 (52.8)	187 (47.3)	33 (33.4)	0.05
Female	1086 (47.3)	697 (47.2)	192 (52.7)	51 (66.6)	
Race/Ethnicity, *n* (%)					
Non-Hispanic White	443 (65.9)	662 (83.4)	221 (89.6)	57 (92.4)	<0.0001
Non-Hispanic Black	482 (11.7)	253 (5.9)	80 (6.6)	15 (4.7)	
Hispanic or other	1006 (22.4)	493 (10.7)	78 (3.8)	12 (3.0)	
Education Level, *n* (%)					
Less than high school	845 (29.3)	541 (23.5)	168 (31.7)	37 (31.3)	0.04
High school or GED	571 (36.5)	427 (36.6)	117 (37.7)	31 (43.2)	
Some college or above	438 (34.1)	385 (39.9)	82 (30.6)	14 (25.5)	
Income-to-poverty ratio (PIR), *n* (%)					
PIR ≤ 1	553 (17.6)	258 (9.6)	54 (8.2)	15 (9.7)	<0.0001
1 < PIR ≤ 3	815 (44.7)	647 (47.3)	164 (38.0)	43 (51.4)	
>3	382 (37.7)	386 (43.1)	126 (53.8)	24 (38.9)	
Smoke status, *n* (%)					
Non-smoker	1045 (47.5)	651 (44.9)	152 (38.9)	40 (46.4)	<0.0001
Former smoker	426 (24.7)	462 (34.4)	163 (44.9)	31 (41.9)	
Current Smoker	459 (27.8)	295 (20.7)	64 (16.2)	13 (11.7)	
Alcohol drinking status, *n* (%)					
Non-drinker	387 (15.3)	235 (14.0)	59 (13.8)	14 (14.5)	0.94
Former drinker	723 (34.8)	537 (36.3)	156 (35.2)	43 (42.3)	
Current drinker	781 (49.9)	614 (49.7)	160 (51.0)	26 (43.2)	
Physical activity *, *n* (%)					0.009
Inactive	536 (19.9)	295 (14.1)	81 (15.2)	31 (29.5)	
Insufficiently active	794 (44.3)	607 (46.3)	174 (46.0)	26 (29.5)	
Recommended activity	600 (35.8)	500 (39.7)	124 (38.8)	27 (41.0)	
Total energy intake (kcal/day), Mean (SE)	2243 (39)	2163 (71)	1988 (73)	1536 (130)	<0.0001
Total daily Mg intake, Mean (SE)	314.5 (6.6)	325.5 (10.1)	323.1 (13.3)	274.2 (15.8)	0.94
BMI (kg/m^2^), Mean (SE)	30.0 (0.4)	30.0 (0.3)	30.5 (0.4)	32.1 (0.8)	0.04
FIB4, Mean (SE)	0.8 (0.0)	1.0 (0.0)	1.2 (0.0)	1.5 (0.1)	<0.0001

Value present as unweighted frequency (weighted percentage, %) or weighted mean (SE). Rao–Scott chi-square test was used for categorical data, and survey regression model for continuous variables. * Inactive denotes no physical activity reported in the last month; insufficiently active denotes those who did not meet the criteria for recommended levels of physical activity in the last month; recommended activity denotes those who met the criteria for recommended physical activity of self-reported leisure time of moderate activity [metabolic equivalent (MET) intensity level for activity ranging from 3 to 6] of five or more times per week or leisure time of vigorous activity (MET > 6) three or more times per week. Abbreviations: BMI, body mass index; FIB4, Fibrosis-4 index; GED, General Education Development; Mg, magnesium; MDS, magnesium depletion score; MASLD, metabolic dysfunction associated steatotic liver disease; MetALD, metabolic and alcohol associated liver disease; NHANES III, the third National Health and Nutrition Examination Survey; SE, standard error.

**Table 2:** Multivariable-adjusted HRs and 95%CI for MDS in relation to all-cause and CVD mortality among participants with MASLD or MetALD in the NHANES III (MASLD + MetALD, *n* = 3802).

HR (95%CI)	MDS				
	0	1	2	>2	
	*n* = 1931	*n* = 1408	*n* = 379	*n* = 84	*p*-Trend
All-cause mortality					
All					
Deaths	549	727	281	81	
Person-years	47,956	30,182	6878	1059	
Model 1	1 (ref)	1.16 (0.92, 1.45)	1.27 (0.97, 1.66)	2.29 (1.56, 3.38)	0.0007
Model 2	1 (ref)	1.23 (0.97, 1.55)	1.43 (1.08, 1.89)	2.44 (1.67, 3.55)	<0.0001
Model 3	1 (ref)	1.27 (1.00, 1.61)	1.48 (1.11, 1.97)	2.52 (1.77, 3.61)	<0.0001
<EAR					
Deaths	336	410	146	43	
Person-years	24,165	14,905	3392	514	
Model 1	1 (ref)	1.23 (0.92, 1.65)	1.20 (0.81, 1.76)	2.35 (1.56, 3.53)	0.02
Model 2	1 (ref)	1.30 (0.94, 1.80)	1.31 (0.90, 1.91)	2.65 (1.70, 4.13)	0.004
Model 3	1 (ref)	1.35 (0.97, 1.89)	1.39 (0.95, 2.01)	2.72 (1.69, 4.37)	0.001
≥EAR					
Deaths	213	317	135	38	
Person-years	23,790	15,276	3485	545	
Model 1	1 (ref)	1.13 (0.85, 1.50)	1.36 (0.97, 1.92)	2.23 (1.33, 3.74)	0.003
Model 2	1 (ref)	1.13 (0.82, 1.54)	1.53 (1.06, 2.21)	2.19 (1.28, 3.73)	0.0006
Model 3	1 (ref)	1.16 (0.83, 1.61)	1.56 (1.07, 2.26)	2.29 (1.36, 3.85)	0.0003
*p*-interaction					0.93
CVD mortality					
All					
Deaths	162	243	104	33	
Person-years	47,956	30,182	6878	1059	
Model 1	1 (ref)	1.37 (1.02, 1.84)	1.43 (0.95, 2.16)	3.07 (1.83, 5.18)	0.0009
Model 2	1 (ref)	1.44 (1.09, 1.89)	1.64 (1.11, 2.43)	3.17 (1.96, 5.15)	<0.0001
Model 3	1 (ref)	1.42 (1.08, 1.87)	1.67 (1.17, 2.39)	3.01 (1.87, 4.86)	<0.0001
<EAR					
Deaths	104	136	55	17	
Person-years	24,165	14,905	3392	514	
Model 1	1 (ref)	1.60 (1.03, 2.49)	1.61 (0.89, 2.90)	3.68 (1.90, 7.15)	0.001
Model 2	1 (ref)	1.70 (1.10, 2.63)	1.74 (1.00, 3.03)	3.64 (1.82, 7.27)	0.0007
Model 3	1 (ref)	1.79 (1.15, 2.80)	1.89 (1.10, 3.28)	3.77 (1.88, 7.55)	0.0004
≥EAR					
Deaths	58	107	49	16	
Person-years	23,790	15,276	3485	545	
Model 1	1 (ref)	1.16 (0.73, 1.85)	1.29 (0.70, 2.38)	2.38 (0.96, 5.89)	0.12
Model 2	1 (ref)	1.24 (0.77, 2.01)	1.58 (0.85, 2.93)	2.58 (0.99, 6.74)	0.03
Model 3	1 (ref)	1.18 (0.72, 1.92)	1.51 (0.84, 2.74)	2.33 (0.86, 6.35)	0.06
*p*-interaction					0.93

Model 1: adjusted for age, sex, and race/ethnicity. Model 2: model 1 + additionally adjusted for education level, ratio of poverty to income, cigarette smoking status, alcohol drinking status, physical activity, BMI, total energy intake, magnesium intake. Model 3: model 2 + additionally adjusted for FIB4. Age- and sex-specific EAR [1] was used to classify magnesium intakes. Abbreviations: BMI, body mass index; CVD, cardiovascular disease; EAR, estimated average requirement; Mg, magnesium; MDS, magnesium depletion score; MASLD, metabolic dysfunction associated steatotic liver disease; MetALD, metabolic and alcohol associated liver disease; NHANES III, the third National Health and Nutrition Examination Survey.

**Table 3:** Multivariable-adjusted HRs and 95%CI for MDS in relation to all-cause and CVD mortality among pure MASLD participants in the NHANES III (pure MASLD, *n* = 3560).

HR (95%CI)	MDS				
	0	1	2	>2	
	*n* = 1923	*n* = 1270	*n* = 296	*n* = 71	*p*-Trend
All-cause mortality					
All					
Deaths	548	692	236	68	
Person-years	47,737	26,752	5079	860	
Model 1	1 (ref)	1.17 (0.92, 1.48)	1.44 (1.09, 1.89)	2.25 (1.37, 3.68)	0.001
Model 2	1 (ref)	1.25 (0.98, 1.60)	1.62 (1.20, 2.21)	2.55 (1.63, 3.99)	<0.0001
Model 3	1 (ref)	1.30 (1.01, 1.67)	1.68 (1.22, 2.32)	2.72 (1.75, 4.22)	<0.0001
<EAR					
Deaths	336	391	123	37	
Person-years	24,139	13,448	2605	419	
Model 1	1 (ref)	1.21 (0.91, 1.62)	1.36 (0.94, 1.96)	2.60 (1.67, 4.04)	0.004
Model 2	1 (ref)	1.27 (0.91, 1.79)	1.44 (0.95, 2.17)	2.89 (1.78, 4.69)	0.004
Model 3	1 (ref)	1.32 (0.93, 1.87)	1.49 (0.97, 2.30)	2.97 (1.74, 5.04)	0.003
≥EAR					
Deaths	212	301	113	31	
Person-years	23,597	13,304	2473	441	
Model 1	1 (ref)	1.17 (0.87, 1.55)	1.54 (1.12, 2.11)	1.96 (1.01, 3.81)	0.004
Model 2	1 (ref)	1.21 (0.89, 1.63)	1.78 (1.24, 2.56)	2.09 (1.15, 3.80)	0.0005
Model 3	1 (ref)	1.24 (0.90, 1.71)	1.82 (1.24, 2.67)	2.24 (1.21, 4.17)	0.0003
*p*-interaction					0.71
CVD mortality					
All					
Deaths	162	235	91	29	
Person-years	47,737	26,752	5079	860	
Model 1	1 (ref)	1.40 (1.03, 1.90)	1.82 (1.17, 2.83)	3.56 (1.98, 6.40)	0.0001
Model 2	1 (ref)	1.49 (1.11, 1.98)	2.16 (1.43, 3.26)	4.00 (2.34, 6.85)	<0.0001
Model 3	1 (ref)	1.47 (1.10, 1.95)	2.19 (1.49, 3.22)	3.86 (2.29, 6.50)	<0.0001
<EAR					
Deaths	104	132	49	15	
Person-years	24,139	13,448	2605	419	
Model 1	1 (ref)	1.65 (1.06, 2.58)	2.15 (1.18, 3.90)	4.66 (2.05, 10.57)	0.0002
Model 2	1 (ref)	1.76 (1.10, 2.80)	2.19 (1.18, 4.05)	4.36 (1.95, 9.75)	0.0002
Model 3	1 (ref)	1.85 (1.14, 3.00)	2.43 (1.31, 4.54)	4.65 (2.10, 10.31)	<0.0001
≥EAR					
Deaths	58	103	42	14	
Person-years	23,597	13,304	2473	441	
Model 1	1 (ref)	1.17 (0.73, 1.89)	1.52 (0.81, 2.87)	2.54 (0.96, 6.71)	0.06
Model 2	1 (ref)	1.34 (0.83, 2.17)	2.23 (1.21, 4.13)	3.40 (1.25, 9.30)	0.004
Model 3	1 (ref)	1.26 (0.78, 2.06)	2.04 (1.12, 3.73)	3.02 (1.05, 8.71)	0.01
*p*-interaction					0.96

Model 1: adjusted for age, sex, and race/ethnicity. Model 2: model 1 + additionally adjusted for education level, ratio of poverty to income, cigarette smoking status, alcohol drinking status, physical activity, BMI, total energy intake, magnesium intake. Model 3: model 2 + additionally adjusted for FIB4. Age- and sex-specific EAR [1] was used to classify magnesium intakes. Abbreviations: BMI, body mass index; CVD, cardiovascular disease; EAR, estimated average requirement; Mg, magnesium; MDS, magnesium depletion score; MASLD, metabolic dysfunction associated steatotic liver disease; NHANES III, the third National Health and Nutrition Examination Survey.

**Table 4:** Stratified analyses by FIB4 index for MDS in relation to all-cause and CVD mortality among participants with MASLD or MetALD in the NHANES III (MASLD + MetALD, *n* = 3802).

HR (95%CI)	MDS				
	0	1	2	>2	
	*n* = 1931	*n* = 1408	*n* = 379	*n* = 84	*p*-Trend
All-cause mortality					
All					
Deaths	549	727	281	81	
Person-years	47,956	30,182	6878	1059	
Model 1	1 (ref)	1.16 (0.92, 1.45)	1.27 (0.97, 1.66)	2.29 (1.56, 3.38)	0.0007
Model 2	1 (ref)	1.23 (0.97, 1.55)	1.43 (1.08, 1.89)	2.44 (1.67, 3.55)	<0.0001
Model 3	1 (ref)	1.27 (1.00, 1.61)	1.48 (1.11, 1.97)	2.52 (1.77, 3.61)	<0.0001
FIB4 < 1.3					
Deaths	370	380	140	36	
Person-years	40,867	22,414	4387	485	
Model 1	1 (ref)	1.13 (0.81, 1.58)	1.37 (0.98, 1.92)	2.82 (1.51, 5.28)	0.004
Model 2	1 (ref)	1.24 (0.89, 1.73)	1.53 (1.10, 2.12)	2.81 (1.63, 4.87)	0.0004
Model 3	1 (ref)	1.25 (0.89, 1.76)	1.47 (1.07, 2.04)	2.95 (1.69, 5.15)	0.0006
FIB4 ≥ 1.3					
Deaths	179	347	141	45	
Person-years	7089	7768	2491	574	
Model 1	1 (ref)	1.22 (0.88, 1.70)	1.18 (0.77, 1.81)	1.94 (1.23, 3.04)	0.08
Model 2	1 (ref)	1.15 (0.82, 1.62)	1.15 (0.72, 1.86)	1.71 (1.07, 2.73)	0.14
Model 3	1 (ref)	1.19 (0.78, 1.83)	1.23 (0.76, 2.00)	1.89 (1.15, 3.10)	0.04
*p*-interaction					0.57
CVD mortality					
All					
Deaths	162	243	104	33	
Person-years	47,956	30,182	6878	1059	
Model 1	1 (ref)	1.37 (1.02, 1.84)	1.43 (0.95, 2.16)	3.07 (1.83, 5.18)	0.0009
Model 2	1 (ref)	1.44 (1.09, 1.89)	1.64 (1.11, 2.43)	3.17 (1.96, 5.15)	<0.0001
Model 3	1 (ref)	1.42 (1.08, 1.87)	1.67 (1.17, 2.39)	3.01 (1.87, 4.86)	<0.0001
FIB4 < 1.3					
Deaths	116	109	51	15	
Person-years	40,867	22,414	4387	485	
Model 1	1 (ref)	1.23 (0.76, 1.99)	1.70 (1.15, 2.53)	3.45 (1.58, 7.50)	0.001
Model 2	1 (ref)	1.27 (0.82, 1.97)	2.01 (1.39, 2.90)	3.21 (1.67, 6.17)	<0.0001
Model 3	1 (ref)	1.31 (0.83, 2.07)	1.92 (1.31, 2.81)	3.38 (1.77, 6.44)	<0.0001
FIB4 ≥ 1.3					
Deaths	46	134	53	18	
Person-years	7089	7768	2491	574	
Model 1	1 (ref)	1.63 (0.90, 2.97)	1.26 (0.67, 2.34)	2.81 (1.24, 6.37)	0.10
Model 2	1 (ref)	1.48 (0.80, 2.74)	1.19 (0.63, 2.26)	2.15 (0.87, 5.33)	0.24
Model 3	1 (ref)	1.36 (0.66, 2.79)	1.15 (0.58, 2.31)	1.92 (0.78, 4.69)	0.32
*p*-interaction					0.32

Model 1: adjusted for age, sex, and race/ethnicity. Model 2: model 1 + additionally adjusted for education level, ratio of poverty to income, cigarette smoking status, alcohol drinking status, physical activity, BMI, total energy intake, magnesium intake. Model 3: model 2 + additionally adjusted for FIB4. Age- and sex-specific EAR [1] was used to classify magnesium intakes. Abbreviations: BMI, body mass index; CVD, cardiovascular disease; EAR, estimated average requirement; FIB4, Fibrosis-4 index; Mg, magnesium; MDS, magnesium depletion score; MASLD, metabolic dysfunction associated steatotic liver disease; MetALD, metabolic and alcohol associated liver disease; NHANES III, the third National Health and Nutrition Examination Survey.

**Table 5:** Stratified analyses by FIB4 index for MDS in relation to all-cause and CVD mortality among pure MASLD participants in the NHANES III (pure MASLD, *n* = 3560).

HR (95%CI)	MDS				
	0	1	2	>2	
	*n* = 1923	*n* = 1270	*n* = 296	*n* = 71	*p*-Trend
All-cause mortality					
All					
Deaths	548	692	236	68	
Person-years	47,737	26,752	5079	860	
Model 1	1 (ref)	1.17 (0.92, 1.48)	1.44 (1.09, 1.89)	2.25 (1.37, 3.68)	0.001
Model 2	1 (ref)	1.25 (0.98, 1.60)	1.62 (1.20, 2.21)	2.55 (1.63, 3.99)	<0.0001
Model 3	1 (ref)	1.30 (1.01, 1.67)	1.68 (1.22, 2.32)	2.72 (1.75, 4.22)	<0.0001
FIB4 < 1.3					
Deaths	369	359	123	30	
Person-years	40,705	19,570	3181	401	
Model 1	1 (ref)	1.16 (0.82, 1.64)	1.70 (1.19, 2.43)	2.59 (1.17, 5.74)	0.004
Model 2	1 (ref)	1.28 (0.90, 1.81)	1.92 (1.29, 2.87)	2.87 (1.47, 5.60)	0.0007
Model 3	1 (ref)	1.29 (0.90, 1.83)	1.84 (1.24, 2.75)	3.03 (1.53, 6.00)	0.001
FIB4 ≥ 1.3					
Deaths	179	333	113	38	
Person-years	7032	7181	1898	459	
Model 1	1 (ref)	1.20 (0.86, 1.67)	1.18 (0.80, 1.76)	1.88 (1.14, 3.10)	0.08
Model 2	1 (ref)	1.12 (0.80, 1.58)	1.13 (0.72, 1.77)	1.56 (0.94, 2.58)	0.20
Model 3	1 (ref)	1.14 (0.74, 1.76)	1.18 (0.72, 1.92)	1.75 (0.98, 3.15)	0.10
*p*-interaction					0.29
CVD mortality					
All					
Deaths	162	235	91	29	
Person-years	47,737	26,752	5079	860	
Model 1	1 (ref)	1.40 (1.03, 1.90)	1.82 (1.17, 2.83)	3.56 (1.98, 6.40)	0.0001
Model 2	1 (ref)	1.49 (1.11, 1.98)	2.16 (1.43, 3.26)	4.00 (2.34, 6.85)	<0.0001
Model 3	1 (ref)	1.47 (1.10, 1.95)	2.19 (1.49, 3.22)	3.86 (2.29, 6.50)	<0.0001
FIB4 < 1.3					
Deaths	116	106	45	13	
Person-years	40,705	19,570	3181	401	
Model 1	1 (ref)	1.29 (0.77, 2.14)	2.13 (1.36, 3.32)	3.73 (1.58, 8.81)	0.0003
Model 2	1 (ref)	1.36 (0.86, 2.15)	2.84 (1.82, 4.44)	3.96 (2.09, 7.47)	<0.0001
Model 3	1 (ref)	1.40 (0.87, 2.24)	2.69 (1.70, 4.27)	4.25 (2.26, 7.99)	<0.0001
FIB4 ≥ 1.3					
Deaths	46	129	46	16	
Person-years	7032	7181	1898	459	
Model 1	1 (ref)	1.65 (0.90, 3.02)	1.65 (0.90, 3.02)	3.26 (1.36, 7.78)	0.01
Model 2	1 (ref)	1.53 (0.79, 2.95)	1.54 (0.80, 2.96)	2.46 (0.82, 7.36)	0.08
Model 3	1 (ref)	1.37 (0.64, 2.96)	1.46 (0.70, 3.08)	2.23 (0.74, 6.73)	0.09
*p*-interaction					0.38

Model 1: adjusted for age, sex, and race/ethnicity. Model 2: model 1 + additionally adjusted for education level, ratio of poverty to income, cigarette smoking status, alcohol drinking status, physical activity, BMI, total energy intake, magnesium intake. Model 3: model 2 + additionally adjusted for FIB4. Age- and sex-specific EAR [1] was used to classify magnesium intakes. Abbreviations: BMI, body mass index; CVD, cardiovascular disease; EAR, estimated average requirement; FIB4, Fibrosis-4 index; Mg, magnesium; MDS, magnesium depletion score; MASLD, metabolic dysfunction associated steatotic liver disease; NHANES III, the third National Health and Nutrition Examination Survey.

## Data Availability

The data presented in this study are openly available from https://wwwn.cdc.gov/nchs/nhanes/Nhanes3/Default.aspx, accessed on 2 December 2023.

## Supplementary Materials

The supporting information can be downloaded at: https://www.mdpi.com/article/10.3390/nu17020244/s1.

## References

[1] Powell E.E., Wong V.W.-S., Rinella M. (2021). Non-Alcoholic Fatty Liver Disease. Lancet.

[2] Younossi Z.M., Koenig A.B., Abdelatif D., Fazel Y., Henry L., Wymer M. (2016). Global Epidemiology of Nonalcoholic Fatty Liver Disease-Meta-Analytic Assessment of Prevalence, Incidence, and Outcomes. Hepatology.

[3] Younossi Z., Anstee Q.M., Marietti M., Hardy T., Henry L., Eslam M., George J., Bugianesi E. (2018). Global Burden of NAFLD and NASH: Trends, Predictions, Risk Factors and Prevention. Nat. Rev. Gastroenterol. Hepatol..

[4] Haflidadottir S., Jonasson J.G., Norland H., Einarsdottir S.O., Kleiner D.E., Lund S.H., Björnsson E.S. (2014). Long-Term Follow-up and Liver-Related Death Rate in Patients with Non-Alcoholic and Alcoholic Related Fatty Liver Disease. BMC Gastroenterol..

[5] Rinella M.E., Lazarus J.V., Ratziu V., Francque S.M., Sanyal A.J., Kanwal F., Romero D., Abdelmalek M.F., Anstee Q.M., Arab J.P. (2023). A Multisociety Delphi Consensus Statement on New Fatty Liver Disease Nomenclature. J. Hepatol..

[6] Moon J.H., Jeong S., Jang H., Koo B.K., Kim W. (2023). Metabolic Dysfunction-Associated Steatotic Liver Disease Increases the Risk of Incident Cardiovascular Disease: A Nationwide Cohort Study. EClinicalMedicine.

[7] Song R., Li Z., Zhang Y., Tan J., Chen Z. (2024). Comparison of NAFLD, MAFLD and MASLD Characteristics and Mortality Outcomes in United States Adults. Liver Int..

[8] Kim D., Wijarnpreecha K., Cholankeril G., Ahmed A. (2024). Metabolic Dysfunction-Associated Steatotic Liver Disease and All-Cause/Cause-Specific Mortality among Adults in the United States. J. Hepatol..

[9] Liu M., Yang H., Mao Y. (2019). Magnesium and Liver Disease. Ann. Transl. Med..

[10] Li W., Zhu X., Song Y., Fan L., Wu L., Kabagambe E.K., Hou L., Shrubsole M.J., Liu J., Dai Q. (2018). Intakes of Magnesium, Calcium and Risk of Fatty Liver Disease and Prediabetes. Public Health Nutr..

[11] Wu L., Zhu X., Fan L., Kabagambe E.K., Song Y., Tao M., Zhong X., Hou L., Shrubsole M.J., Liu J. (2017). Magnesium Intake and Mortality Due to Liver Diseases: Results from the Third National Health and Nutrition Examination Survey Cohort. Sci. Rep..

[12] Emamat H., Ghalandari H., Totmaj A.S., Tangestani H., Hekmatdoost A. (2021). Calcium to Magnesium Intake Ratio and Non-Alcoholic Fatty Liver Disease Development: A Case-Control Study. BMC Endocr. Disord..

[13] Lu L., Chen C., Li Y., Guo W., Zhang S., Brockman J., Shikany J.M., Kahe K. (2022). Magnesium Intake Is Inversely Associated with Risk of Non-Alcoholic Fatty Liver Disease among American Adults. Eur. J. Nutr..

[14] Tao M.-H., Fulda K.G. (2021). Association of Magnesium Intake with Liver Fibrosis among Adults in the United States. Nutrients.

[15] Yu Y.-C., Paragomi P., Wang R., Liang F., Luu H.N., Behari J., Yuan J.-M. (2023). High Serum Magnesium Is Associated with Lower Risk of Hepatocellular Carcinoma among Patients with Nonalcoholic Fatty Liver Disease. Cancer.

[16] Fan L., Zhu X., Rosanoff A., Costello R.B., Yu C., Ness R., Seidner D.L., Murff H.J., Roumie C.L., Shrubsole M.J. (2021). Magnesium Depletion Score (MDS) Predicts Risk of Systemic Inflammation and Cardiovascular Mortality among US Adults. J. Nutr..

[17] Fiorentini D., Cappadone C., Farruggia G., Prata C. (2021). Magnesium: Biochemistry, Nutrition, Detection, and Social Impact of Diseases Linked to Its Deficiency. Nutrients.

[18] Manual P. (1988). Third National Health and Nutrition Examination Survey. https://pdfs.semanticscholar.org/97a8/2d927cd2b59e821a97013c24f59255f86419.pdf.

[19] Chan W.-K., Chuah K.-H., Rajaram R.B., Lim L.-L., Ratnasingam J., Vethakkan S.R. (2023). Metabolic Dysfunction-Associated Steatotic Liver Disease (MASLD): A State-of-the-Art Review. J. Obes. Metab. Syndr..

[20] Tian Z., Qu S., Yana C., Fang J., Song X., He K., Jiang K., Sun X., Shi J., Tao Y. (2024). Associations of the Magnesium Depletion Score and Magnesium Intake with Diabetes among US Adults: An Analysis of the National Health and Nutrition Examination Survey 2011–2018. Epidemiol. Health.

[21] Tan M.-Y., Mo C.-Y., Zhao Q. (2023). The Association between Magnesium Depletion Score and Hypertension in US Adults: Evidence from the National Health and Nutrition Examination Survey (2007–2018). Biol. Trace Elem. Res..

[22] Xu Y., Qin Y., Lu H., Liu L., Huang W., Huang A., Ye Y., Shen H., Guo Z., Chen W. (2024). The Magnesium Depletion Score Is Associated with Increased Likelihood of Kidney Stone Disease among Female Adults. J. Trace Elem. Med. Biol..

[23] Wang X., Zeng Z., Wang X., Zhao P., Xiong L., Liao T., Yuan R., Yang S., Kang L., Liang Z. (2024). Magnesium Depletion Score and Metabolic Syndrome in US Adults: Analysis of NHANES 2003 to 2018. J. Clin. Endocrinol. Metab..

[24] Yin S., Zhou Z., Lin T., Wang X. (2022). Magnesium Depletion Score Is Associated with Long-Term Mortality in Chronic Kidney Diseases: A Prospective Population-Based Cohort Study. J. Nephrol..

[25] Zhao D., Chen P., Chen M., Chen L., Wang L. (2023). Association of Magnesium Depletion Score with Congestive Heart Failure: Results from the NHANES 2007–2016. Biol. Trace Elem. Res..

[26] Ye L., Zhang C., Duan Q., Shao Y., Zhou J. (2023). Association of Magnesium Depletion Score with Cardiovascular Disease and Its Association with Longitudinal Mortality in Patients with Cardiovascular Disease. J. Am. Heart Assoc..

[27] Chen Y., Xiang X., Wu Y., Han S., Huang Z., Wu M. (2022). Magnesium Depletion Score Predicts Diabetic Retinopathy Risk among Diabetes: Findings from NHANES 2005–2018. Biol. Trace Elem. Res..

[28] Wang J., Xing F., Sheng N., Xiang Z. (2022). Associations of the Dietary Magnesium Intake and Magnesium Depletion Score With Osteoporosis Among American Adults: Data From the National Health and Nutrition Examination Survey. Front. Nutr..

[29] Lu J., Li H., Wang S. (2022). The Kidney Reabsorption-Related Magnesium Depletion Score Is Associated with Increased Likelihood of Abdominal Aortic Calcification among US Adults. Nephrol. Dial. Transplant..

[30] Arnold M.J., Harding M.C., Conley A.T. (2021). Dietary Guidelines for Americans 2020–2025: Recommendations from the US Departments of Agriculture and Health and Human Services. Am. Fam. Physician.

[31] https://www.niaaa.nih.gov/alcohol-health/overview-alcohol-consumption/moderate-binge-drinking.

[32] Alcohol Research: Current Reviews Editorial Staff (2018). Drinking Patterns and Their Definitions. Alcohol. Res..

[33] NHANES 2003–2004: Dietary Interview-Individual Foods, First Day Data Documentation, Codebook, and Frequencies. https://wwwn.cdc.gov/Nchs/Data/Nhanes/Public/2003/DataFiles/DR1IFF_C.htm.

[34] Institute of Medicine (US) Standing Committee on the Scientific Evaluation of Dietary Reference Intakes (1997). Dietary Reference Intakes for Calcium, Phosphorus, Magnesium, Vitamin D, and Fluoride.

[35] Vallet-Pichard A., Mallet V., Nalpas B., Verkarre V., Nalpas A., Dhalluin-Venier V., Fontaine H., Pol S. (2007). FIB-4: An Inexpensive and Accurate Marker of Fibrosis in HCV Infection. Comparison with Liver Biopsy and Fibrotest. Hepatology.

[36] Portillo-Sanchez P., Bril F., Maximos M., Lomonaco R., Biernacki D., Orsak B., Subbarayan S., Webb A., Hecht J., Cusi K. (2015). High Prevalence of Nonalcoholic Fatty Liver Disease in Patients with Type 2 Diabetes Mellitus and Normal Plasma Aminotransferase Levels. J. Clin. Endocrinol. Metab..

[37] Tacke F., Horn P., Wai-Sun Wong V., Ratziu V., Bugianesi E., Francque S., Zelber-Sagi S., Valenti L., Roden M., Schick F. (2024). EASL–EASD–EASO Clinical Practice Guidelines on the Management of Metabolic Dysfunction-Associated Steatotic Liver Disease (MASLD). J. Hepatol..

[38] Kwak M., Kim H.-S., Jiang Z.G., Yeo Y.H., Trivedi H.D., Noureddin M., Yang J.D. (2024). MASLD/MetALD and Mortality in Individuals with Any Cardio-Metabolic Risk Factor: A Population-Based Study with 26.7 Years of Follow-Up. Hepatology.

[39] Barbagallo M., Veronese N., Dominguez L.J. (2022). Magnesium in Type 2 Diabetes Mellitus, Obesity, and Metabolic Syndrome. Nutrients.

[40] Piuri G., Zocchi M., Della Porta M., Ficara V., Manoni M., Zuccotti G.V., Pinotti L., Maier J.A., Cazzola R. (2021). Magnesium in Obesity, Metabolic Syndrome, and Type 2 Diabetes. Nutrients.

[41] Gommers L.M.M., Hoenderop J.G.J., Bindels R.J.M., de Baaij J.H.F. (2016). Hypomagnesemia in Type 2 Diabetes: A Vicious Circle?. Diabetes.

[42] Nielsen F.H. (2018). Magnesium Deficiency and Increased Inflammation: Current Perspectives. J. Inflamm. Res..

[43] Ashique S., Kumar S., Hussain A., Mishra N., Garg A., Gowda B.H.J., Farid A., Gupta G., Dua K., Taghizadeh-Hesary F. (2023). A Narrative Review on the Role of Magnesium in Immune Regulation, Inflammation, Infectious Diseases, and Cancer. J. Health Popul. Nutr..

[44] Bagheri A., Naghshi S., Sadeghi O., Larijani B., Esmaillzadeh A. (2021). Total, Dietary, and Supplemental Magnesium Intakes and Risk of All-Cause, Cardiovascular, and Cancer Mortality: A Systematic Review and Dose–Response Meta-Analysis of Prospective Cohort Studies. Adv. Nutr..

[45] Zhang X., Xia J., Del Gobbo L.C., Hruby A., Dai Q., Song Y. (2018). Serum Magnesium Concentrations and All-Cause, Cardiovascular, and Cancer Mortality among U.S. Adults: Results from the NHANES I Epidemiologic Follow-up Study. Clin. Nutr..

[46] Ferraioli G., Soares Monteiro L.B. (2019). Ultrasound-Based Techniques for the Diagnosis of Liver Steatosis. World J. Gastroenterol..

[47] Draijer L.G., Feddouli S., Bohte A.E., Vd Baan Slootweg O., Pels Rijcken T.H., Benninga M.A., Stoker J., Koot B.G.P. (2019). Comparison of Diagnostic Accuracy of Screening Tests ALT and Ultrasound for Pediatric Non-Alcoholic Fatty Liver Disease. Eur. J. Pediatr..

[48] Acharya U.R., Faust O., Molinari F., Sree S.V., Junnarkar S.P., Sudarshan V. (2015). Ultrasound-Based Tissue Characterization and Classification of Fatty Liver Disease: A Screening and Diagnostic Paradigm. Knowl.-Based Syst..

