# Supplementary material for: Magnesium Depletion Score and Mortality in Individuals with Metabolic Dysfunction Associated Steatotic Liver Disease over a Median Follow-Up of 26 Years
Source: Nutrients. 2025 Jan 10;17(2):244. doi: 10.3390/nu17020244 (PMC11767615; doi:10.3390/nu17020244)
Supplement: Supplementary file 1 [file nutrients-17-00244-s001.zip › nutrients-3381869-supplementary.pdf]

**Supplemental materials**

# Magnesium depletion score and mortality in individuals with metabolic dysfunction-associated steatotic liver disease over a median follow-up of 26 years

# Contents

Supplemental Table S1 Baseline demographic and selected risk factors by MDS in pure MASLD participants in the NHANES III (pure MASLD, n=3,560)........................................ 3

Supplemental Table S2 Sensitivity analyses by excluding individuals who died within the first year of follow-up, or with CVD history at baseline, or with cancer history at baseline in participants with MASLD or MetALD and stratified by EAR in the NHANES III (MASLD+MetALD)........................................................................................ 5

Supplemental Table S3 Sensitivity analyses by excluding individuals who died within the first year of follow-up, or with CVD history at baseline, or with cancer history at baseline among pure MASLD participants and stratified by EAR of Mg intake in the NHANES III........ 8

Supplemental Table S4 Sensitivity analyses by excluding individuals who died within the first year of follow-up, or with CVD history at baseline, or with cancer history at baseline in participants with MASLD or MetALD and stratified by FIB4 in the NHANES III (MASLD+MetALD)........................................................................................11

Supplemental Table S5 Sensitivity analyses by excluding individuals who died within the first year of follow-up, or with CVD history at baseline, or with cancer history at baseline among pure MASLD participants and stratified by FIB4 in the NHANES III........................14

Supplemental Table S6 Sensitivity analyses by additionally adjusting for comorbidity of obesity, diabetes, hypertension, dyslipidemia among participants with MASLD or MetALD in the NHANES III (MASLD+MetALD, *n*=3,802) ...............................................................17

Supplemental Table S7 Sensitivity analyses by additionally adjusting for comorbidity of obesity, diabetes, hypertension, dyslipidemia among pure MASLD participants in the NHANES III (pure MASLD, *n*=3,560) ......................................................................18

Supplemental Table S8 Multivariable-adjusted HRs and 95% CI for MDS in relation to cancer mortality among participants with MASLD or MetALD in the NHANES III (MASLD+MetALD, *n*=3,802)...........................................................................................19

Supplemental Table S9 Multivariable-adjusted HRs and 95% CI for MDS in relation to cancer mortality among pure MASLD participants in the NHANES III (pure MASLD, *n*=3,560) ........................................................................................................................20

Supplemental Table S1 Baseline demographic and selected risk factors by MDS in pure MASLD participants in the NHANES III (pure MASLD, n=3,560)

| **Characteristics** | **MDS=0** | **MDS=1** | **MDS=2** | **MDS>2** | ***P* value** |
|---|---|---|---|---|---|
| | *n*=1,923 | *n*=1,270 | *n*=296 | *n*=71 | |
| Age (year), Mean (SD) | 39.7 (0.4) | 50.8 (0.8) | 60.9 (0.8) | 64.3 (1.3) | <0.0001 |
| Gender, n (%) | | | | | |
| Male | 840 (52.9) | 620 (51.3) | 126 (37.7) | 29 (37.2) | 0.004 |
| Female | 1083 (47.1) | 650 (48.7) | 170 (62.3) | 42 (62.8) | |
| Race/Ethnicity, n (%) | | | | | |
| Non-Hispanic White | 441 (65.9) | 628 (84.5) | 169 (88.3) | 44 (90.0) | <0.0001 |
| Non-Hispanic Black | 481 (11.7) | 219 (5.5) | 68 (8.3) | 15 (6.1) | |
| Hispanic or other | 1001 (22.5) | 423 (10.0) | 59 (3.3) | 12 (3.9) | |
| Education Level, n (%) | | | | | |
| Less Than High School | 842 (29.4) | 484 (22.5) | 138 (35.3) | 34 (35.3) | 0.002 |
| High School or GED | 569 (36.6) | 386 (37.2) | 89 (37.7) | 25 (42.6) | |
| Some College or Above | 435 (34.0) | 349 (40.3) | 59 (27.0) | 10 (22.1) | |
| Income-to-poverty ratio (PIR), n (%) | | | | | |
| PIR≤1 | 553 (17.7) | 230 (9.5) | 48 (9.0) | 14 (10.5) | 0.0002 |
| 1<PIR≤3 | 809 (44.5) | 573 (46.3) | 137 (46.2) | 38 (52.9) | |
| >3 | 382 (37.8) | 362 (44.2) | 85 (44.8) | 17 (36.5) | |
| Smoke status, n (%) | | | | | |
| None-smoker | 1042 (47.5) | 610 (46.2) | 131 (42.7) | 37 (50.1) | <0.0001 |
| Former smoker | 423 (24.6) | 428 (34.8) | 129 (46.0) | 28 (47.0) | |
| Current Smoker | 457 (27.9) | 232 (19.0) | 36 (11.3) | 6 (2.9) | |
| Alcohol drinking status, n (%) | | | | | |
| None-drinker | 386 (15.3) | 234 (15.1) | 59 (20.1) | 14 (19.0) | 0.0001 |
| Former drinker | 717 (34.6) | 535 (39.4) | 156 (51.2) | 43 (55.7) | |
| Current drinker | 781 (50.1) | 480 (45.5) | 77 (28.7) | 13 (25.2) | |
| Physical activity*, n (%) | | | | | 0.002 |

| | | | | | |
|---|---|---|---|---|---|
| Inactive | 534 (20.0) | 268 (13.6) | 74 (20.1) | 29 (35.2) | |
| Insufficiently active | 792 (44.4) | 548 (46.7) | 128 (44.7) | 22 (32.5) | |
| Recommended activity | 596 (35.6) | 450 (39.7) | 94 (35.1) | 20 (32.3) | |
| Total energy intake (kcal/day), Mean (SD) | 2242 (39) | 2104 (76) | 1837 (75) | 1512 (122) | <0.0001 |
| Total daily Mg intake, Mean (SD) | 314.5 (6.6) | 322.2 (10.5) | 303.3 (13.9) | 269.2 (16.2) | 0.4858 |
| BMI (kg/m$^2$), Mean (SD) | 30.0 (0.4) | 30.3 (0.3) | 31.7 (0.5) | 32.6 (1.1) | 0.0003 |
| FIB4, Mean (SD) | 0.8 (0.0) | 1.1 (0.0) | 1.3 (0.1) | 1.4 (0.1) | <0.0001 |

Value present as unweighted frequency (weighted percentage, %) or weighted mean (SD). Rao-Scott chi-square test for categorical data, and survey regression model for continuous variables. *Inactive denotes no physical activity reported in the last month; insufficiently active denotes those who did not meet the criteria for recommended levels of physical activity in the last month; recommended activity denotes those who met the criteria for recommended physical activity of self-reported leisure time moderate activity [metabolic equivalent (MET) intensity level for activity ranging from 3 to 6] of five or more times per week or leisure time vigorous activity (MET>6) three or more times per week. Abbreviations: BMI, body mass index; FIB4, Fibrosis-4 index; GED, General Education Development; Mg, magnesium; MDS, magnesium depletion score; MASLD, metabolic dysfunction-associated steatotic liver disease; MetALD, metabolic and alcohol related/associated liver disease; NHANES III, the third National Health and Nutrition Examination Survey.

Supplemental Table S2 Sensitivity analyses by excluding individuals who died within the first year of follow-up, or with CVD history at baseline, or with cancer history at baseline in participants with MASLD or MetALD and stratified by EAR in the NHANES III (MASLD+MetALD)

| | MDS | | | | |
|---|---|---|---|---|---|
| **HR (95% CI)** | **0** | **1** | **2** | **>2** | ***P*-trend** |
| All-cause mortality | Exclude individuals who died within the first year of follow-up (*n*=3,794) | | | | |
| All | *n*=1,929 | *n*=1,405 | *n*=379 | *n*=81 | |
| Deaths | 547 | 724 | 281 | 78 | |
| Person-years | 47,954 | 30,179 | 6,878 | 1,056 | |
| Model 1 | 1 (ref) | 1.16(0.92,1.45) | 1.28(0.98,1.67) | 2.17(1.48,3.17) | 0.0012 |
| Model 2 | 1 (ref) | 1.23(0.97,1.55) | 1.43(1.08,1.90) | 2.30(1.59,3.34) | <.0001 |
| Model 3 | 1 (ref) | 1.26(0.99,1.61) | 1.49(1.11,1.98) | 2.38(1.68,3.38) | <.0001 |
| <EAR | | | | | |
| Deaths | 334 | 407 | 146 | 43 | |
| Person-years | 24,163 | 14,903 | 3,393 | 514 | |
| Model 1 | 1 (ref) | 1.23(0.91,1.64) | 1.20(0.81,1.77) | 2.36(1.57,3.57) | 0.0208 |
| Model 2 | 1 (ref) | 1.30(0.94,1.80) | 1.32(0.91,1.92) | 2.67(1.71,4.18) | 0.0033 |
| Model 3 | 1 (ref) | 1.35(0.97,1.88) | 1.40(0.96,2.03) | 2.75(1.70,4.43) | 0.0012 |
| ≥EAR | | | | | |
| Deaths | 213 | 317 | 135 | 35 | |
| Person-years | 23,790 | 15,276 | 3,485 | 542 | |
| Model 1 | 1 (ref) | 1.13(0.85,1.50) | 1.36(0.97,1.92) | 1.96(1.19,3.25) | 0.0071 |
| Model 2 | 1 (ref) | 1.13(0.82,1.55) | 1.54(1.07,2.21) | 1.90(1.11,3.25) | 0.0024 |
| Model 3 | 1 (ref) | 1.15(0.83,1.61) | 1.56(1.07,2.27) | 1.98(1.18,3.33) | 0.0014 |
| *P*-interaction | | | | | 0.82 |
| CVD mortality | Exclude individuals with CVD history at baseline (*n*=3,592) | | | | |
| All | *n*=1,877 | *n*=1,316 | *n*=331 | *n*=68 | |
| Deaths | 145 | 205 | 83 | 25 | |

| | | | | | |
|---|---|---|---|---|---|
| Person-years | 46,961 | 28,811 | 6,259 | 938 | |
| Model 1 | 1 (ref) | 1.46(1.11,1.92) | 1.49(0.99,2.24) | 2.62(1.43,4.81) | 0.0068 |
| Model 2 | 1 (ref) | 1.52(1.15,2.02) | 1.71(1.14,2.57) | 2.72(1.56,4.74) | 0.0009 |
| Model 3 | 1 (ref) | 1.52(1.14,2.01) | 1.74(1.21,2.50) | 2.54(1.46,4.40) | 0.0007 |
| <EAR | | | | | |
| Deaths | 92 | 107 | 42 | 14 | |
| Person-years | 23,639 | 14,002 | 3,009 | 431 | |
| Model 1 | 1 (ref) | 1.55(1.01,2.38) | 1.57(0.88,2.81) | 3.41(1.61,7.23) | 0.0083 |
| Model 2 | 1 (ref) | 1.72(1.08,2.73) | 1.81(0.97,3.39) | 3.69(1.69,8.09) | 0.0054 |
| Model 3 | 1 (ref) | 1.84(1.16,2.90) | 2.01(1.09,3.71) | 3.96(1.84,8.49) | 0.0022 |
| ≥EAR | | | | | |
| Deaths | 53 | 98 | 41 | 11 | |
| Person-years | 23,321 | 14,809 | 3,249 | 507 | |
| Model 1 | 1 (ref) | 1.40(0.93,2.11) | 1.48(0.80,2.74) | 1.94(0.76,4.94) | 0.1689 |
| Model 2 | 1 (ref) | 1.41(0.89,2.22) | 1.82(0.95,3.48) | 2.03(0.76,5.47) | 0.0611 |
| Model 3 | 1 (ref) | 1.32(0.82,2.11) | 1.71(0.91,3.20) | 1.68(0.57,4.92) | 0.1191 |
| *P*-interaction | | | | | 0.91 |
| Cancer mortality | Exclude individuals with cancer history at baseline (*n*=3,689) | | | | |
| All | *n*=1,892 | *n*=1,354 | *n*=367 | *n*=76 | |
| Deaths | 118 | 151 | 54 | 8 | |
| Person-years | 47,137 | 29,262 | 6,726 | 972 | |
| Model 1 | 1 (ref) | 1.04(0.65,1.67) | 1.06(0.63,1.77) | 1.33(0.53,3.35) | 0.6433 |
| Model 2 | 1 (ref) | 1.09(0.67,1.78) | 1.19(0.70,2.01) | 1.40(0.55,3.58) | 0.3915 |
| Model 3 | 1 (ref) | 1.10(0.67,1.83) | 1.22(0.70,2.12) | 1.43(0.59,3.45) | 0.3296 |
| <EAR | | | | | |
| Deaths | 74 | 79 | 29 | 6 | |
| Person-years | 23,776 | 14,472 | 3,307 | 483 | |
| Model 1 | 1 (ref) | 0.86(0.49,1.52) | 0.89(0.41,1.93) | 1.73(0.67,4.50) | 0.8374 |
| Model 2 | 1 (ref) | 0.80(0.43,1.47) | 0.93(0.42,2.06) | 1.93(0.65,5.78) | 0.7508 |

| | | | | | |
|---|---|---|---|---|---|
| Model 3 | 1 (ref) | 0.73(0.37,1.44) | 0.88(0.39,1.96) | 1.74(0.56,5.38) | 0.8687 |
| ≥EAR | | | | | |
| Deaths | 44 | 72 | 25 | 2 | |
| Person-years | 23,361 | 14,789 | 3,418 | 489 | |
| Model 1 | 1 (ref) | 1.27(0.63,2.53) | 1.23(0.57,2.64) | 0.64(0.11,3.73) | 0.7677 |
| Model 2 | 1 (ref) | 1.36(0.67,2.75) | 1.40(0.65,3.02) | 0.61(0.11,3.50) | 0.6031 |
| Model 3 | 1 (ref) | 1.53(0.70,3.33) | 1.61(0.76,3.40) | 0.74(0.15,3.57) | 0.304 |
| *P*-interaction | | | | | 0.68 |

Model 1: adjusted for age, sex, and race/ethnicity.
Model 2: model 1+additionally adjusted for education level, ratio of poverty to income, cigarette smoking status, alcohol drinking status, physical activity, BMI, total energy intake, magnesium intake.
Model 3: model 2+additionally adjusted for FIB4.
Age- and sex-specific EAR[36] was used to classify magnesium intakes.
Abbreviations: BMI, body mass index; CVD, cardiovascular disease; EAR, estimated average requirement; Mg, magnesium; MDS, magnesium depletion score; MASLD, metabolic dysfunction-associated steatotic liver disease; MetALD, metabolic and alcohol related/associated liver disease; NHANES III, the third National Health and Nutrition Examination Survey.

Supplemental Table S3 Sensitivity analyses by excluding individuals who died within the first year of follow-up, or with CVD history at baseline, or with cancer history at baseline among pure MASLD participants and stratified by EAR of Mg intake in the NHANES III

| | MDS | | | | |
|---|---|---|---|---|---|
| **HR (95% CI)** | **0** | **1** | **2** | **>2** | ***P*-trend** |
| All-cause mortality | Exclude individuals who died within the first year of follow-up (*n*=3,552) | | | | |
| All | *n*=1,921 | *n*=1,267 | *n*=296 | *n*=68 | |
| Deaths | 546 | 689 | 236 | 65 | |
| Person-years | 47,734 | 26,749 | 5,078 | 857 | |
| Model 1 | 1 (ref) | 1.17(0.92,1.48) | 1.44(1.09,1.90) | 2.07(1.28,3.37) | 0.0021 |
| Model 2 | 1 (ref) | 1.25(0.98,1.60) | 1.63(1.20,2.22) | 2.35(1.52,3.65) | 0.0002 |
| Model 3 | 1 (ref) | 1.30(1.00,1.67) | 1.69(1.22,2.34) | 2.50(1.63,3.83) | <.0001 |
| <EAR | | | | | |
| Deaths | 334 | 388 | 123 | 37 | |
| Person-years | 24,137 | 13,445 | 2,605 | 418 | |
| Model 1 | 1 (ref) | 1.21(0.90,1.62) | 1.37(0.94,1.97) | 2.61(1.67,4.08) | 0.0035 |
| Model 2 | 1 (ref) | 1.27(0.91,1.78) | 1.45(0.96,2.19) | 2.93(1.80,4.76) | 0.0033 |
| Model 3 | 1 (ref) | 1.31(0.93,1.87) | 1.50(0.98,2.32) | 3.01(1.76,5.13) | 0.0023 |
| ≥EAR | | | | | |
| Deaths | 212 | 301 | 113 | 28 | |
| Person-years | 23,597 | 13,304 | 2,473 | 438 | |
| Model 1 | 1 (ref) | 1.17(0.88,1.56) | 1.54(1.12,2.11) | 1.62(0.86,3.04) | 0.0091 |
| Model 2 | 1 (ref) | 1.21(0.89,1.64) | 1.78(1.23,2.57) | 1.69(0.95,3.00) | 0.0025 |
| Model 3 | 1 (ref) | 1.24(0.90,1.72) | 1.82(1.24,2.68) | 1.79(0.99,3.25) | 0.0015 |
| *P*-interaction | | | | | 0.47 |
| CVD mortality | Exclude individuals with CVD history at baseline (*n*=3,353) | | | | |
| All | *n*=1,869 | *n*=1,180 | *n*=248 | *n*=56 | |
| Deaths | 145 | 197 | 70 | 21 | |
| Person-years | 46,741 | 25,426 | 4,459 | 747 | |

| | | | | | |
|---|---|---|---|---|---|
| Model 1 | 1 (ref) | 1.49(1.12,1.99) | 1.91(1.24,2.94) | 2.81(1.39,5.67) | 0.0008 |
| Model 2 | 1 (ref) | 1.58(1.17,2.13) | 2.28(1.47,3.52) | 3.19(1.67,6.09) | <.0001 |
| Model 3 | 1 (ref) | 1.57(1.16,2.12) | 2.29(1.54,3.42) | 2.99(1.62,5.53) | <.0001 |
| <EAR | | | | | |
| Deaths | 92 | 103 | 36 | 12 | |
| Person-years | 23,613 | 12,589 | 2,222 | 344 | |
| Model 1 | 1 (ref) | 1.60(1.04,2.47) | 2.18(1.20,3.95) | 4.12(1.76,9.66) | 0.0007 |
| Model 2 | 1 (ref) | 1.81(1.09,3.00) | 2.48(1.21,5.05) | 4.45(1.78,11.11) | 0.0013 |
| Model 3 | 1 (ref) | 1.94(1.16,3.24) | 2.83(1.40,5.74) | 5.00(2.08,12.03) | 0.0003 |
| ≥EAR | | | | | |
| Deaths | 53 | 98 | 41 | 11 | |
| Person-years | 23,321 | 14,809 | 3,249 | 507 | |
| Model 1 | 1 (ref) | 1.41(0.92,2.17) | 1.73(0.90,3.31) | 1.83(0.63,5.35) | 0.1149 |
| Model 2 | 1 (ref) | 1.52(0.95,2.44) | 2.51(1.26,4.97) | 2.25(0.75,6.76) | 0.0135 |
| Model 3 | 1 (ref) | 1.42(0.87,2.31) | 2.23(1.13,4.40) | 1.69(0.48,5.88) | 0.0491 |
| *P*-interaction | | | | | 0.74 |
| Cancer mortality | | Exclude individuals with cancer history at baseline (*n*=3,451) | | | |
| All | *n*=1,884 | *n*=1,217 | *n*=285 | *n*=65 | |
| Deaths | 118 | 141 | 45 | 6 | |
| Person-years | 46,918 | 25,860 | 4,943 | 814 | |
| Model 1 | 1 (ref) | 1.01(0.61,1.67) | 1.13(0.66,1.92) | 1.10(0.31,3.96) | 0.73 |
| Model 2 | 1 (ref) | 1.09(0.64,1.85) | 1.35(0.76,2.37) | 1.34(0.36,5.06) | 0.3793 |
| Model 3 | 1 (ref) | 1.10(0.63,1.92) | 1.36(0.76,2.41) | 1.47(0.40,5.39) | 0.3306 |
| <EAR | | | | | |
| Deaths | 74 | 73 | 24 | 5 | |
| Person-years | 23,750 | 13,014 | 2,520 | 409 | |
| Model 1 | 1 (ref) | 0.76(0.43,1.35) | 0.83(0.42,1.66) | 1.70(0.55,5.20) | 0.9767 |
| Model 2 | 1 (ref) | 0.69(0.37,1.29) | 0.88(0.39,2.01) | 1.92(0.49,7.51) | 0.9329 |
| Model 3 | 1 (ref) | 0.63(0.31,1.26) | 0.79(0.34,1.81) | 1.74(0.43,7.01) | 0.9218 |

| | | | | | |
|---|---|---|---|---|---|
| ≥EAR | | | | | |
| Deaths | 44 | 68 | 21 | 1 | |
| Person-years | 23,168 | 12,845 | 2,423 | 404 | |
| Model 1 | 1 (ref) | 1.31(0.66,2.62) | 1.47(0.71,3.02) | 0.14(0.02,1.11) | 0.626 |
| Model 2 | 1 (ref) | 1.49(0.73,3.02) | 1.71(0.76,3.82) | 0.15(0.02,1.33) | 0.4117 |
| Model 3 | 1 (ref) | 1.70(0.79,3.64) | 1.89(0.90,3.96) | 0.21(0.02,1.86) | 0.1617 |
| *P*-interaction | | | | | 0.73 |

Model 1: adjusted for age, sex, and race/ethnicity.
Model 2: model 1+additionally adjusted for education level, ratio of poverty to income, cigarette smoking status, alcohol drinking status, physical activity, BMI, total energy intake, magnesium intake.
Model 3: model 2+additionally adjusted for FIB4.
Age- and sex-specific EAR[36] was used to classify magnesium intakes.
Abbreviations: BMI, body mass index; CVD, cardiovascular disease; EAR, estimated average requirement; Mg, magnesium; MDS, magnesium depletion score; MASLD, metabolic dysfunction-associated steatotic liver disease; NHANES III, the third National Health and Nutrition Examination Survey.

Supplemental Table S4 Sensitivity analyses by excluding individuals who died within the first year of follow-up, or with CVD history at baseline, or with cancer history at baseline in participants with MASLD or MetALD and stratified by FIB4 in the NHANES III (MASLD+MetALD)

| | MDS | | | | |
|---|---|---|---|---|---|
| **HR (95% CI)** | **0** | **1** | **2** | **>2** | ***P*-trend** |
| All-cause mortality | Exclude individuals who died within the first year of follow-up (*n*=3,794) | | | | |
| All | *n*=1,929 | *n*=1,405 | *n*=379 | *n*=81 | |
| Deaths | 547 | 724 | 281 | 78 | |
| Person-years | 47,954 | 30,179 | 6,878 | 1,056 | |
| Model 1 | 1 (ref) | 1.16(0.92,1.45) | 1.28(0.98,1.67) | 2.17(1.48,3.17) | 0.0012 |
| Model 2 | 1 (ref) | 1.23(0.97,1.55) | 1.43(1.08,1.90) | 2.30(1.59,3.34) | <.0001 |
| Model 3 | 1 (ref) | 1.26(0.99,1.61) | 1.49(1.11,1.98) | 2.38(1.68,3.38) | <.0001 |
| FIB4<1.3 | | | | | |
| Deaths | 369 | 379 | 140 | 35 | |
| Person-years | 40,866 | 22,413 | 4,386 | 484 | |
| Model 1 | 1 (ref) | 1.14(0.81,1.59) | 1.38(0.98,1.93) | 2.75(1.49,5.05) | 0.0038 |
| Model 2 | 1 (ref) | 1.24(0.89,1.74) | 1.54(1.11,2.13) | 2.75(1.62,4.65) | 0.0004 |
| Model 3 | 1 (ref) | 1.25(0.89,1.76) | 1.48(1.07,2.05) | 2.89(1.69,4.93) | 0.0006 |
| FIB4≥1.3 | | | | | |
| Deaths | 178 | 345 | 141 | 43 | |
| Person-years | 7,087 | 7,766 | 2,491 | 572 | |
| Model 1 | 1 (ref) | 1.22(0.87,1.69) | 1.18(0.77,1.81) | 1.77(1.14,2.77) | 0.1416 |
| Model 2 | 1 (ref) | 1.14(0.80,1.61) | 1.14(0.70,1.86) | 1.53(0.93,2.50) | 0.2675 |
| Model 3 | 1 (ref) | 1.17(0.75,1.81) | 1.21(0.74,1.99) | 1.67(1.03,2.72) | 0.0997 |
| *P*-interaction | | | | | 0.50 |
| CVD mortality | Exclude individuals with CVD history at baseline (*n*=3,592) | | | | |
| All | *n*=1,877 | *n*=1,316 | *n*=331 | *n*=68 | |
| Deaths | 145 | 205 | 83 | 25 | |

| | | | | | |
|---|---|---|---|---|---|
| Person-years | 46,961 | 28,811 | 6,259 | 938 | |
| Model 1 | 1 (ref) | 1.46(1.11,1.92) | 1.49(0.99,2.24) | 2.62(1.43,4.81) | 0.0068 |
| Model 2 | 1 (ref) | 1.52(1.15,2.02) | 1.71(1.14,2.57) | 2.72(1.56,4.74) | 0.0009 |
| Model 3 | 1 (ref) | 1.52(1.14,2.01) | 1.74(1.21,2.50) | 2.54(1.46,4.40) | 0.0007 |
| FIB4<1.3 | | | | | |
| Deaths | 107 | 97 | 40 | 12 | |
| Person-years | 40,191 | 21,690 | 4,016 | 430 | |
| Model 1 | 1 (ref) | 1.35(0.85,2.15) | 1.77(1.17,2.68) | 3.14(1.30,7.57) | 0.0013 |
| Model 2 | 1 (ref) | 1.43(0.91,2.24) | 2.04(1.35,3.07) | 2.80(1.35,5.83) | <.0001 |
| Model 3 | 1 (ref) | 1.45(0.92,2.31) | 1.96(1.27,3.00) | 2.92(1.47,5.82) | 0.0001 |
| FIB4≥1.3 | | | | | |
| Deaths | 38 | 108 | 43 | 13 | |
| Person-years | 6,769 | 7,120 | 2,242 | 508 | |
| Model 1 | 1 (ref) | 1.70(0.86,3.36) | 1.30(0.66,2.57) | 2.24(0.92,5.48) | 0.2888 |
| Model 2 | 1 (ref) | 1.47(0.73,2.99) | 1.22(0.58,2.57) | 1.47(0.52,4.21) | 0.6928 |
| Model 3 | 1 (ref) | 1.39(0.61,3.15) | 1.21(0.53,2.76) | 1.28(0.47,3.48) | 0.9062 |
| *P*-interaction | | | | | 0.40 |
| Cancer mortality | Exclude individuals with cancer history at baseline (*n*=3,689) | | | | |
| All | *n*=1,892 | *n*=1,354 | *n*=367 | *n*=76 | |
| Deaths | 118 | 151 | 54 | 8 | |
| Person-years | 47,138 | 29,262 | 6,726 | 973 | |
| Model 1 | 1 (ref) | 1.04(0.65,1.67) | 1.06(0.63,1.77) | 1.33(0.53,3.35) | 0.6433 |
| Model 2 | 1 (ref) | 1.09(0.67,1.78) | 1.19(0.70,2.01) | 1.40(0.55,3.58) | 0.3915 |
| Model 3 | 1 (ref) | 1.10(0.67,1.83) | 1.22(0.70,2.12) | 1.43(0.59,3.45) | 0.3296 |
| FIB4<1.3 | | | | | |
| Deaths | 82 | 85 | 29 | 5 | |
| Person-years | 40,167 | 21,841 | 4,253 | 445 | |
| Model 1 | 1 (ref) | 0.84(0.48,1.46) | 1.20(0.66,2.17) | 1.64(0.47,5.74) | 0.5657 |
| Model 2 | 1 (ref) | 0.90(0.50,1.61) | 1.27(0.74,2.19) | 1.42(0.40,5.07) | 0.4546 |

| | | | | | |
|---|---|---|---|---|---|
| Model 3 | 1 (ref) | 0.90(0.50,1.63) | 1.24(0.73,2.10) | 1.43(0.39,5.18) | 0.4879 |
| FIB4≥1.3 | | | | | |
| Deaths | 36 | 66 | 25 | 3 | |
| Person-years | 6,970 | 7,420 | 2,473 | 527 | |
| Model 1 | 1 (ref) | 1.61(0.75,3.44) | 1.07(0.39,2.93) | 1.48(0.39,5.68) | 0.8241 |
| Model 2 | 1 (ref) | 1.24(0.55,2.80) | 0.94(0.32,2.73) | 1.25(0.25,6.14) | 0.9933 |
| Model 3 | 1 (ref) | 1.17(0.51,2.69) | 0.91(0.33,2.47) | 1.51(0.48,4.78) | 0.9434 |
| *P*-interaction | | | | | 0.47 |

Model 1: adjusted for age, sex, and race/ethnicity.
Model 2: model 1+additionally adjusted for education level, ratio of poverty to income, cigarette smoking status, alcohol drinking status, physical activity, BMI, total energy intake, magnesium intake.
Model 3: model 2+additionally adjusted for FIB4.
Age- and sex-specific EAR[36] was used to classify magnesium intakes.
Abbreviations: BMI, body mass index; CVD, cardiovascular disease; EAR, estimated average requirement; FIB4, Fibrosis-4 index; Mg, magnesium; MDS, magnesium depletion score; MASLD, metabolic dysfunction-associated steatotic liver disease; MetALD, metabolic and alcohol related/associated liver disease; NHANES III, the third National Health and Nutrition Examination Survey.

Supplemental Table S5 Sensitivity analyses by excluding individuals who died within the first year of follow-up, or with CVD history at baseline, or with cancer history at baseline among pure MASLD participants and stratified by FIB4 in the NHANES III

| | MDS | | | | |
|---|---|---|---|---|---|
| **HR (95% CI)** | **0** | **1** | **2** | **>2** | ***P*-trend** |
| All-cause mortality | Exclude individuals who died within the first year of follow-up (*n*=3,552) | | | | |
| All | *n*=1,921 | *n*=1,267 | *n*=296 | *n*=68 | |
| Deaths | 546 | 689 | 236 | 65 | |
| Person-years | 47,734 | 26,749 | 5,078 | 857 | |
| Model 1 | 1 (ref) | 1.17(0.92,1.48) | 1.44(1.09,1.90) | 2.07(1.28,3.37) | 0.0021 |
| Model 2 | 1 (ref) | 1.25(0.98,1.60) | 1.63(1.20,2.22) | 2.35(1.52,3.65) | 0.0002 |
| Model 3 | 1 (ref) | 1.30(1.00,1.67) | 1.69(1.22,2.34) | 2.50(1.63,3.83) | <.0001 |
| FIB4<1.3 | | | | | |
| Deaths | 368 | 358 | 123 | 29 | |
| Person-years | 40,704 | 19,569 | 3,181 | 400 | |
| Model 1 | 1 (ref) | 1.16(0.82,1.65) | 1.71(1.20,2.45) | 2.49(1.15,5.38) | 0.004 |
| Model 2 | 1 (ref) | 1.28(0.90,1.82) | 1.94(1.29,2.90) | 2.77(1.47,5.21) | 0.0006 |
| Model 3 | 1 (ref) | 1.29(0.90,1.84) | 1.86(1.24,2.78) | 2.92(1.52,5.61) | 0.001 |
| FIB4≥1.3 | | | | | |
| Deaths | 178 | 331 | 113 | 36 | |
| Person-years | 7,030 | 7,179 | 1,898 | 457 | |
| Model 1 | 1 (ref) | 1.19(0.85,1.67) | 1.18(0.79,1.76) | 1.67(1.03,2.73) | 0.151 |
| Model 2 | 1 (ref) | 1.10(0.78,1.57) | 1.10(0.69,1.76) | 1.33(0.80,2.22) | 0.4269 |
| Model 3 | 1 (ref) | 1.11(0.72,1.73) | 1.15(0.69,1.90) | 1.48(0.85,2.58) | 0.2395 |
| *P*-interaction | | | | | 0.24 |
| CVD mortality | Exclude individuals with CVD history at baseline (*n*=3,353) | | | | |
| All | *n*=1,869 | *n*=1,180 | *n*=248 | *n*=56 | |
| Deaths | 145 | 197 | 70 | 21 | |

| | | | | | |
|---|---|---|---|---|---|
| Person-years | 46,741 | 25,426 | 4,459 | 747 | |
| Model 1 | 1 (ref) | 1.49(1.12,1.99) | 1.91(1.24,2.94) | 2.81(1.39,5.67) | 0.0008 |
| Model 2 | 1 (ref) | 1.58(1.17,2.13) | 2.28(1.47,3.52) | 3.19(1.67,6.09) | <.0001 |
| Model 3 | 1 (ref) | 1.57(1.16,2.12) | 2.29(1.54,3.42) | 2.99(1.62,5.53) | <.0001 |
| FIB4<1.3 | | | | | |
| Deaths | 107 | 94 | 34 | 10 | |
| Person-years | 40,029 | 18,892 | 2,810 | 353 | |
| Model 1 | 1 (ref) | 1.43(0.87,2.33) | 2.20(1.40,3.45) | 2.98(1.05,8.42) | 0.0006 |
| Model 2 | 1 (ref) | 1.54(0.97,2.46) | 2.89(1.83,4.56) | 2.97(1.32,6.67) | <.0001 |
| Model 3 | 1 (ref) | 1.57(0.97,2.53) | 2.76(1.72,4.43) | 3.18(1.48,6.86) | <.0001 |
| FIB4≥1.3 | | | | | |
| Deaths | 38 | 103 | 36 | 11 | |
| Person-years | 6,712 | 6,533 | 1,649 | 393 | |
| Model 1 | 1 (ref) | 1.70(0.85,3.39) | 1.74(0.87,3.47) | 2.45(0.88,6.81) | 0.063 |
| Model 2 | 1 (ref) | 1.48(0.69,3.20) | 1.63(0.74,3.60) | 1.50(0.39,5.71) | 0.3704 |
| Model 3 | 1 (ref) | 1.35(0.56,3.25) | 1.60(0.66,3.90) | 1.26(0.34,4.70) | 0.4716 |
| *P*-interaction | | | | | 0.41 |
| Cancer mortality | Exclude individuals with cancer history at baseline (*n*=3,451) | | | | |
| All | *n*=1,884 | *n*=1,217 | *n*=285 | *n*=65 | |
| Deaths | 118 | 141 | 45 | 6 | |
| Person-years | 46,918 | 25,860 | 4,943 | 814 | |
| Model 1 | 1 (ref) | 1.01(0.61,1.67) | 1.13(0.66,1.92) | 1.10(0.31,3.96) | 0.73 |
| Model 2 | 1 (ref) | 1.09(0.64,1.85) | 1.35(0.76,2.37) | 1.34(0.36,5.06) | 0.3793 |
| Model 3 | 1 (ref) | 1.10(0.63,1.92) | 1.36(0.76,2.41) | 1.47(0.40,5.39) | 0.3306 |
| FIB4<1.3 | | | | | |
| Deaths | 82 | 77 | 25 | 5 | |
| Person-years | 40,005 | 18,998 | 3,064 | 361 | |
| Model 1 | 1 (ref) | 0.78(0.44,1.40) | 1.26(0.74,2.14) | 2.03(0.58,7.12) | 0.5269 |
| Model 2 | 1 (ref) | 0.82(0.43,1.59) | 1.40(0.80,2.46) | 2.19(0.54,8.80) | 0.3703 |

| | | | | | |
|---|---|---|---|---|---|
| Model 3 | 1 (ref) | 0.83(0.43,1.60) | 1.36(0.78,2.38) | 2.21(0.55,8.87) | 0.4 |
| FIB4≥1.3 | | | | | |
| Deaths | 36 | 64 | 20 | 1 | |
| Person-years | 6,913 | 6,861 | 1,879 | 454 | |
| Model 1 | 1 (ref) | 1.66(0.75,3.68) | 1.16(0.37,3.69) | 0.58(0.07,4.64) | 0.9503 |
| Model 2 | 1 (ref) | 1.27(0.54,2.98) | 1.08(0.29,4.11) | 0.45(0.05,4.39) | 0.8787 |
| Model 3 | 1 (ref) | 1.18(0.51,2.74) | 1.07(0.31,3.77) | 0.61(0.07,5.02) | 0.9036 |
| *P*-interaction | | | | | 0.16 |

Model 1: adjusted for age, sex, and race/ethnicity.
Model 2: model 1+additionally adjusted for education level, ratio of poverty to income, cigarette smoking status, alcohol drinking status, physical activity, BMI, total energy intake, magnesium intake.
Model 3: model 2+additionally adjusted for FIB4.
Age- and sex-specific EAR[36] was used to classify magnesium intakes.
Abbreviations: BMI, body mass index; CVD, cardiovascular disease; EAR, estimated average requirement; FIB4, Fibrosis-4 index; Mg, magnesium; MDS, magnesium depletion score; MASLD, metabolic dysfunction-associated steatotic liver disease; MetALD, metabolic and alcohol related/associated liver disease; NHANES III, the third National Health and Nutrition Examination Survey.

Supplemental Table S6 Sensitivity analyses by additionally adjusting for comorbidity of obesity, diabetes, hypertension, dyslipidemia among participants with MASLD or MetALD in the NHANES III (MASLD+MetALD, *n*=3,802)

| | **MDS** | | | | |
|---|---|---|---|---|---|
| | **0** | **1** | **2** | **>2** | |
| **HR (95% CI)** | *n*=1,931 | *n*=1,408 | *n*=379 | *n*=84 | ***P*-trend** |
| All-cause mortality | | | | | |
| Deaths | 549 | 727 | 281 | 81 | |
| Person-years | 47,956 | 30,182 | 6,878 | 1,059 | |
| Model 4 | 1 (ref) | 1.23(0.95,1.59) | 1.40(1.03,1.88) | 2.07(1.47,2.91) | 0.0005 |
| CVD mortality | | | | | |
| Deaths | 162 | 243 | 104 | 33 | |
| Person-years | 47,956 | 30,182 | 6,878 | 1,059 | |
| Model 4 | 1 (ref) | 1.35(0.97,1.87) | 1.56(1.08,2.25) | 2.21(1.32,3.68) | 0.0019 |
| Cancer mortality | | | | | |
| Deaths | 129 | 160 | 59 | 12 | |
| Person-years | 47,956 | 30,182 | 6,878 | 1,059 | |
| Model 4 | 1 (ref) | 1.05(0.63,1.75) | 1.20(0.69,2.09) | 1.65(0.68,4.03) | 0.2873 |

Model 4: in addition to model 3 (adjusted for age, sex, race/ethnicity, education level, ratio of poverty to income, cigarette smoking status, alcohol drinking status, physical activity, BMI, total energy intake, magnesium intake, FIB4) + additionally adjusted for comorbidity of obesity, diabetes, hypertension, dyslipidemia.

Abbreviations: BMI, body mass index; CVD, cardiovascular disease; MDS, magnesium depletion score; MASLD, metabolic dysfunction-associated steatotic liver disease; MetALD, metabolic and alcohol related/associated liver disease; NHANES III, the third National Health and Nutrition Examination Survey.

Supplemental Table S7 Sensitivity analyses by additionally adjusting for comorbidity of obesity, diabetes, hypertension, dyslipidemia among pure MASLD participants in the NHANES III (pure MASLD, *n*=3,560)

| | MDS | | | | |
|---|---|---|---|---|---|
| | **0** | **1** | **2** | **>2** | |
| **HR (95% CI)** | *n*=1,923 | *n*=1,270 | *n*=296 | *n*=71 | ***P*-trend** |
| All-cause mortality | | | | | |
| Deaths | 548 | 692 | 236 | 68 | |
| Person-years | 47,737 | 26,752 | 5,079 | 860 | |
| Model 4 | 1 (ref) | 1.26(0.96,1.65) | 1.52(1.08,2.14) | 2.27(1.46,3.52) | 0.0007 |
| CVD mortality | | | | | |
| Deaths | 162 | 235 | 91 | 29 | |
| Person-years | 47,737 | 26,752 | 5,079 | 860 | |
| Model 4 | 1 (ref) | 1.38(0.99,1.93) | 1.84(1.20,2.80) | 2.94(1.73,5.01) | <.0001 |
| Cancer mortality | | | | | |
| Deaths | 129 | 150 | 50 | 9 | |
| Person-years | 47,737 | 26,752 | 5,079 | 860 | |
| Model 4 | 1 (ref) | 1.05(0.60,1.82) | 1.39(0.80,2.41) | 1.90(0.49,7.43) | 0.2767 |

Model 4: in addition to model 3 (adjusted for age, sex, race/ethnicity, education level, ratio of poverty to income, cigarette smoking status, alcohol drinking status, physical activity, BMI, total energy intake, magnesium intake, FIB4) + additionally adjusted for comorbidity of obesity, diabetes, hypertension, dyslipidemia.

Abbreviations: BMI, body mass index; CVD, cardiovascular disease; MDS, magnesium depletion score; MASLD, metabolic dysfunction-associated steatotic liver disease; NHANES III, the third National Health and Nutrition Examination Survey.

Supplemental Table S8 Multivariable-adjusted HRs and 95% CI for MDS in relation to cancer mortality among participants with MASLD or MetALD in the NHANES III (MASLD+MetALD, *n*=3,802)

| | MDS | | | | |
|---|---|---|---|---|---|
| | **0** | **1** | **2** | **>2** | |
| **HR (95% CI)** | *n*=1,931 | *n*=1,408 | *n*=379 | *n*=84 | ***P*-trend** |
| Cancer mortality | | | | | |
| All | | | | | |
| Deaths | 129 | 160 | 59 | 12 | |
| Person-years | 47,956 | 30,182 | 6,878 | 1,059 | |
| Model 1 | 1 (ref) | 0.96(0.60,1.56) | 1.02(0.62,1.69) | 1.52(0.56,4.17) | 0.65 |
| Model 2 | 1 (ref) | 1.02(0.61,1.69) | 1.17(0.70,1.97) | 1.59(0.56,4.54) | 0.41 |
| Model 3 | 1 (ref) | 1.08(0.66,1.76) | 1.22(0.71,2.11) | 1.72(0.67,4.37) | 0.26 |
| <EAR | | | | | |
| Deaths | 81 | 84 | 33 | 8 | |
| Person-years | 24,165 | 14,905 | 3,392 | 514 | |
| Model 1 | 1 (ref) | 0.83(0.46,1.51) | 0.94(0.46,1.93) | 1.71(0.63,4.63) | 0.78 |
| Model 2 | 1 (ref) | 0.78(0.41,1.50) | 0.99(0.43,2.25) | 1.74(0.55,5.47) | 0.73 |
| Model 3 | 1 (ref) | 0.78(0.42,1.47) | 0.92(0.41,2.06) | 1.70(0.59,4.88) | 0.77 |
| ≥EAR | | | | | |
| Deaths | 48 | 76 | 26 | 4 | |
| Person-years | 23,790 | 15,276 | 3,485 | 545 | |
| Model 1 | 1 (ref) | 1.14(0.59,2.20) | 1.11(0.53,2.33) | 1.14(0.28,4.58) | 0.74 |
| Model 2 | 1 (ref) | 1.17(0.61,2.25) | 1.30(0.62,2.72) | 1.01(0.23,4.54) | 0.57 |
| Model 3 | 1 (ref) | 1.35(0.65,2.81) | 1.54(0.74,3.19) | 1.29(0.32,5.12) | 0.23 |
| *P*-interaction | | | | | 0.72 |

Model 1: adjusted for age, sex, and race/ethnicity.
Model 2: model 1+additionally adjusted for education level, ratio of poverty to income, cigarette smoking status, alcohol drinking status, physical activity, BMI, total energy intake, magnesium intake.
Model 3: model 2+additionally adjusted for FIB4.
Age- and sex-specific EAR was used to classify magnesium intakes.
Abbreviations: BMI, body mass index; CVD, cardiovascular disease; EAR, estimated average requirement; Mg, magnesium; MDS, magnesium depletion score; MASLD, metabolic dysfunction-associated steatotic liver disease;

MetALD, metabolic and alcohol related/associated liver disease; NHANES III, the third National Health and Nutrition Examination Survey.

Supplemental Table S9 Multivariable-adjusted HRs and 95% CI for MDS in relation to cancer mortality among pure MASLD participants in the NHANES III (pure MASLD, *n*=3,560)

| | MDS | | | | |
|---|---|---|---|---|---|
| | **0** | **1** | **2** | **>2** | |
| **HR (95% CI)** | *n*=1,923 | *n*=1,270 | *n*=296 | *n*=71 | ***P*-trend** |
| Cancer mortality | | | | | |
| All | | | | | |
| Deaths | 129 | 150 | 50 | 9 | |
| Person-years | 47,737 | 26,752 | 5,079 | 860 | |
| Model 1 | 1 (ref) | 0.94(0.57,1.55) | 1.12(0.66,1.90) | 1.33(0.32,5.56) | 0.72 |
| Model 2 | 1 (ref) | 1.01(0.58,1.75) | 1.34(0.75,2.39) | 1.58(0.35,7.24) | 0.43 |
| Model 3 | 1 (ref) | 1.08(0.64,1.82) | 1.38(0.79,2.42) | 1.84(0.45,7.50) | 0.28 |
| <EAR | | | | | |
| Deaths | 81 | 78 | 28 | 6 | |
| Person-years | 24,139 | 13,448 | 2,605 | 419 | |
| Model 1 | 1 (ref) | 0.75(0.41,1.36) | 0.91(0.47,1.77) | 1.53(0.46,5.08) | 0.99 |
| Model 2 | 1 (ref) | 0.68(0.35,1.32) | 0.94(0.40,2.21) | 1.62(0.37,7.06) | 0.96 |
| Model 3 | 1 (ref) | 0.68(0.35,1.31) | 0.85(0.38,1.91) | 1.63(0.42,6.38) | 0.98 |
| ≥EAR | | | | | |
| Deaths | 48 | 72 | 22 | 3 | |
| Person-years | 23,597 | 13,304 | 2,473 | 441 | |
| Model 1 | 1 (ref) | 1.18(0.61,2.27) | 1.34(0.67,2.68) | 0.93(0.13,6.42) | 0.55 |
| Model 2 | 1 (ref) | 1.29(0.67,2.46) | 1.62(0.74,3.53) | 0.96(0.12,7.75) | 0.38 |
| Model 3 | 1 (ref) | 1.49(0.73,3.06) | 1.85(0.89,3.88) | 1.33(0.17,10.53) | 0.14 |
| *P*-interaction | | | | | 0.68 |

Model 1: adjusted for age, sex, and race/ethnicity.
Model 2: model 1+additionally adjusted for education level, ratio of poverty to income, cigarette smoking status, alcohol drinking status, physical activity, BMI, total energy intake, magnesium intake.

Model 3: model 2+additionally adjusted for FIB4.
Age- and sex-specific EAR was used to classify magnesium intakes.
Abbreviations: BMI, body mass index; CVD, cardiovascular disease; EAR, estimated average requirement; Mg, magnesium; MDS, magnesium depletion score; MASLD, metabolic dysfunction-associated steatotic liver disease; NHANES III, the third National Health and Nutrition Examination Survey.
